# Edge-Computing Architectures for Internet of Things Applications: A Survey

**DOI:** 10.3390/s20226441

**Published:** 2020-11-11

**Authors:** Salam Hamdan, Moussa Ayyash, Sufyan Almajali

**Affiliations:** 1 Department of Computer Science, Princess Sumaya University for Technology, Amman 11941, Jordan; s.hamdan@psut.edu.jo; 2 Department of Computing, Information, and Mathematical Sciences and Technologies, Chicago State University, Chicago, IL 60628, USA; msma@ieee.org

**Keywords:** Internet of Things, cloud computing, edge computing

## Abstract

The rapid growth of the Internet of Things (IoT) applications and their interference with our daily life tasks have led to a large number of IoT devices and enormous sizes of IoT-generated data. The resources of IoT devices are limited; therefore, the processing and storing IoT data in these devices are inefficient. Traditional cloud-computing resources are used to partially handle some of the IoT resource-limitation issues; however, using the resources in cloud centers leads to other issues, such as latency in time-critical IoT applications. Therefore, edge-cloud-computing technology has recently evolved. This technology allows for data processing and storage at the edge of the network. This paper studies, in-depth, edge-computing architectures for IoT (ECAs-IoT), and then classifies them according to different factors such as data placement, orchestration services, security, and big data. Besides, the paper studies each architecture in depth and compares them according to various features. Additionally, ECAs-IoT is mapped according to two existing IoT layered models, which helps in identifying the capabilities, features, and gaps of every architecture. Moreover, the paper presents the most important limitations of existing ECAs-IoT and recommends solutions to them. Furthermore, this survey details the IoT applications in the edge-computing domain. Lastly, the paper recommends four different scenarios for using ECAs-IoT by IoT applications.

## 1. Introduction

The Internet of Things (IoT) is expanding into different aspects of our lives with technologies and applications in, for example, smart cities, healthcare, and smart homes [1]. Tens of billions of objects are connected to the Internet [2], and the industry expects 50 billion IoT devices to existing by 2020 [3]. However, IoT devices are limited in resources such as storage and processing power, which impacts the performance, security, reliability, and privacy of IoT-based solutions and applications [4,5]. Many applications are enhanced by integrating the IoT and cloud computing. Examples of such applications are in healthcare [6,7,8], smart cities [8,9,10], smart homes [11], smart metering [5], video surveillance [5] such as smart urban surveillance applications [12], agriculture [13], such as greenhouse environment-monitoring system [14], and smart mobility [15], such as smart tourism destinations [16]. Although IoT devices are limited in resources, cloud computing helps IoT in addressing such limitations [5,17].

IoT data are transmitted continuously from applications to a central storage unit, which is usually located in a cloud center [18]. Some IoT applications require low latency time and they may need real-time processing. Handling such requirements by cloud computing is not suitable [19]. Thus, edge computing is crucial for fulfilling these requirements by deploying cloud-computing-like capabilities at the edge of the network [20]. This survey paper focuses on current edge-computing architectures (ECAs) for IoT applications (ECAs-IoT). This paper introduces the ECA-IoT concept and surveys current ECAs-IoT and possible research opportunities. We define an ECA-IoT as a computing architecture that comprises IoT, edge, and cloud-computing devices, software, network protocols, and infrastructure that are connected to deliver certain services. The IoT devices include smart devices, sensors, and actuators that interact and communicate with each other along with end-users. IoT devices are managed and secured by devices that are located at the edge of the network, edge devices, which are under centralized control located in the cloud. ECAs-IoT were proposed to support IoT applications. While some ECAs-IoT focus on solving the data-placement issue to reduce latency time, other ECAs-IoT focus on reducing cost by proposing service-allocation-based architectures. Some ECAs-IoT also focuses on network management by using software-defined networking for network orchestration. Additionally, several ECAs-IoT aims to analyze the huge amount of data that are generated by IoT devices. Other architectures focus on preserving security and privacy. The continuous data transmission from IoT devices increases pressure on bandwidth consumption, which requires efficient approaches for data management, which is the focus of some ECAs-IoT. The majority of IoT surveys handle various aspects, such as IoT architectures, IoT applications, and fog-computing challenges for IoT. However, none of them provided a study on edge computing architectures that handle IoT challenges and classify them. Because many researchers studied Edge computing for a while, incorporating ECAs-IoT and IoT challenges should be of further research.

This paper was written to help in recognizing many open research questions that can be addressed using current ECAs-IoT. Examples of these open research areas include the need for better IoT data-placement strategies inappropriate edge nodes, the need for task allocation among edge nodes to enhance efficiency, the lack of architecture orchestration, and security requirements.

We classified ECAs-IoT according to the following criteria:

- Challenge-based: an ECA-IoT that handles different challenges such as optimizing data placement, task and service allocation, service orchestration, and big-data analysis.
- Technology-based: an ECA-IoT is selected based on the technology it employs, such as software-defined networking (SDN) and machine learning (ML).

In addition to providing an overview of edge-computing technology with a specific focus on edge computing for IoT, this survey extends the current literature in the following ways:

- A new important term (ECA-IoT) that relates key computing technologies (edge, cloud, and IoT) is introduced and defined.
- Existing ECAs-IoT are reviewed in detail. To the best of our knowledge, this is the first survey that classifies them based on IoT challenges. This classification is a starting point for studies that aim to propose new ECAs-IoT.
- Classified ECAs-IoT is mapped to two IoT layered models that are currently used in the literature. This standardization and mapping effort helps in identifying the capabilities, features, and gaps of every architecture.
- Taxonomy is presented for IoT applications that are based on several categories such as the application function, the structure of IoT application, the traffic amount, sensitivity to delay, and the need for data processing at the edge of the network or in the cloud.
- Four new different scenarios for using the ECAs-IoT by IoT applications are recommended. The proposed scenarios are referred to as Use, Modify, Merge, and New.

**Figure 1:**
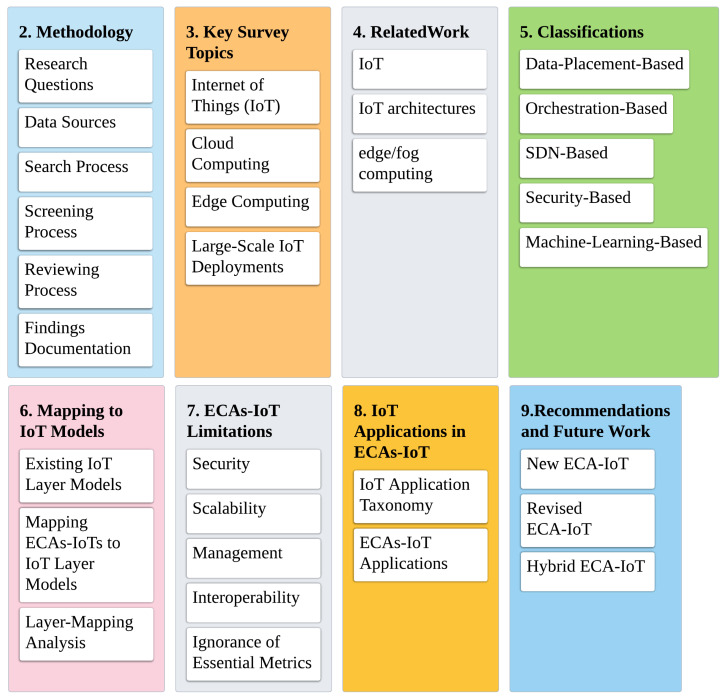
The road map of this review paper.

Figure 1 shows the overall road map of the paper. This paper is organized, as follows. Section 2 provides the methodology of writing this survey. Section 3 provides the background of key survey topics. Section 4 summarizes surveys related to this survey. Section 5 classifies ECAs-IoT on the basis of issues that they address. Section 6 maps ECAs-IoT to 5/3-layer models. Section 7 lists some ECAs-IoT limitations. Section 8.2 classifies and recommends suitable ECAs-IoT for IoT applications. Lastly, Section 10 concludes the paper.

## 2. Methodology

To conduct a systematic review, this article covers the ECAs-IoT. The process of selecting research articles, including the inclusion or exclusion of related articles, is illustrated in the following sections:

### 2.1. Research Questions

The first step is to identify the research questions (RQs), Several factors were considered in selecting the questions, such as IoT application type and their requirements from edge computing, the different IoT applications’ needs, different edge-computing architectures, and their core competency features. The following questions were selected to illuminate the incorporation between ECAs-IoT and IoT challenges for IoT applications:

- RQ1: How can edge computing serve IoT applications?
- RQ2: What are the ECAs-IoT that handle IoT challenges and serve IoT applications? (Section 5)
- RQ3: What is the most appropriate network architecture that can be adopted in ECAs-IoT? (Section 6)
- RQ4: What are the main challenges that ECAS-IoT face? (Section 7)

### 2.2. Data Sources

The initial search was done in five major repositories: IEEE Explore, ACM Digital Library, Elsevier, Springer, and Google Scholar. Most related articles were found in IEEE Explore, which was the main data source of this study. The initial browsing for related articles was to answer RQ1; then, we found that a great number of IoT challenges were covered by ECAs-IoT.

### 2.3. Search Process

To ensure that the extracted articles were recent, most of the literature articles were written between 2014 and 2020. Efficient search keywords were used; these keywords cover how edge computing serves the IoT in many fields, such as IoT applications and challenges. At the same time, keywords considered edge-computing challenges and IoT challenges were separated to avoid missing related key research work. The search keywords were:

- edge computing for IoT applications;
- edge-computing architectures for IoT;
- fog-computing architectures for IoT;
- IoT challenges;
- edge-computing challenges; and,
- IoT application.

The search found more than 1300 articles, and the most relevant articles were chosen.

### 2.4. Screening Process

The duplicated articles were eliminated, and the first step of the screening process was conducted on 520 articles, i.e., checking article titles and keywords for relatedness to this literature review article. This resulted in removing 232 articles. Thereafter, the titles and abstracts of the reaming articles were reviewed, and that resulted in removing unrelated articles. This process eliminated 108 articles. The remaining articles were assessed by studying their content. This assessment focused on the research questions that this article addresses.

### 2.5. Reviewing Process

The previous step left almost 180 articles to be carefully studied and reviewed. To include or exclude articles, we used the following criteria:

- C1: Is the contribution of this article significant?
- C2: Is this article well-structured?
- C3: Are the techniques in the article well-presented?

### 2.6. Findings Documentation

The following sections show the findings of this article. We classified ECAs-IoT on the basis of IoT issues that they addressed and classified IoT applications in ECAs-IoT (RQ1 and RQ2). We also mapped ECAs-IoT to a 5/3-layer model (RQ3). Finally, we classified current ECAs-IoT limitations (RQ4).

## 3. Key Survey Topics

This section covers the necessary background about important concepts mentioned in this paper: IoT network, cloud computing, edge computing, and edge intelligence aspects.

### 3.1. Internet of Things (IoT)

An IoT network is a system of associated and diverse devices, for example, vehicles and home appliances with abilities, such as communication and data transfer over the network. These connected devices overpass the gap between the physical and digital worlds to enhance the quality of life, social life, and industries [21]. Billions of devices all over the world are connected to the Internet, and generate, collect, and transmit data [22]. The potential of connecting anything to the Internet is very broad [23]. Daily life objects could be equipped with networking, sensing, and identifying capabilities [24]. Following are some of the technologies that are broadly deployed in order to support IoT-based services and products [25]:

- Radio-frequency identification (RFID): RFID is a wireless communication technology that automatically identifies and traces objects that are attached to RFID enabled tags [26]. RFID tags vary in storage capabilities according to application requirements. RFID tags have larger storage capabilities when compared with other tracking technologies such as barcode technology [25].
- Wireless-sensor networks (WSNs): a WSN is an infrastructure-less network that consists of scattered devices that are equipped with sensing capabilities to monitor physical and environmental conditions [23].
- Middleware: is an intermediary software that lies between IoT applications and IoT devices [27]. It acts as the bridge between IoT devices and IoT applications. It links various IoT applications with heterogeneous IoT devices for developing, integrating, managing, and communicating over various network interfaces [28].
- Cloud computing: a technology that transforms various services, such as storage, management, and data processing to remote servers [29]. Cloud computing enhances IoT networks by providing efficient online management, storage, and data processing. Cloud computing is further discussed in this paper.

Despite the potential advantages, the IoT faces various challenges in relation to:

- Scalability: the capability of a network, process, or system to deal with an expanding amount of work in a skillful manner. This is a serious issue in the IoT due to the variety of applications accessing raw data [30]. Additionally, many IoT applications involve a large number of IoT devices that continuously change and grow according to need such as smart cities IoT enabled environment.
- Self-organizabiliy: in the case of network failure that happen due to a node, link, or communication failure [31], the network should be able to bootstrap communication between devices. Self-organizing improves network availability [32].
- Data size: the generated data from IoT devices are huge and heterogeneous; therefore, there is a need for an adequate storage mechanism and effective data-transmission protocols in IoT networks [33].
- Timely data analysis: for real-time applications, immediate data analysis is required. Therefore, new technologies, such as edge computing, should be fused with IoT networks [34].
- Interoperability: the heterogeneity and the large number of IoT devices with different functionalities and applications, produced by different trademarks with their own proprietary standards, is a clear challenge. Therefore, IoT networks must be able to deal with heterogeneity and variety.
- Bandwidth scarcity: IoT devices consume bandwidth to collect and transmit data and the increasing number of IoT devices increases the demand for bandwidth; in addition, bandwidth must be available to deal with IoT application requirements [35].

### 3.2. Cloud Computing

Cloud computing is the technology of delivering on-demand computing services over the Internet for servers, databases, and storage through applications and web-based tools [29]. Cloud computing deployment models are classified into four types models [36]: **Public cloud**: this cloud infrastructure provides services for public users over the Internet. This infrastructure is operated, owned, and managed by one or more cloud service providers [36]. **Private cloud**: this cloud infrastructure provides services for a specific usage through a single organization including various consumers. This infrastructure is operated, owned, and managed by the organization [36]. **Community cloud**: this infrastructure is used to provide services to specific usage by a specific consumer from different organizations that have shared concerns, for example, different schools use the same platform that is offered by some organization [25]. **Hybrid cloud**: is a combination of one or more cloud service models. Each model remains unique. For example, merging between a public cloud, such as Google, with a private cloud, such as Amazon web services (AWS). However, they are bonded together by policies that allow for data and application portability [36].

Cloud computing provides services over the Internet. These services are categorized into three categories depending on the provided benefits: **Software-as-a-service (SaaS)**: in this model, vendors provide end-users with a software or an application, mainly via a browser, to do and store their work online [37,38]. **Platform-as-a-service (PaaS)**: vendors provide end-users with platforms that allow the end-user to deploy and run new applications without the need to construct or maintain the entire infrastructure [38]. **Infrastructure-as-a-service (IaaS)**: also known as hardware-as-a-service, vendors provide the consumer with hardware, data centers, network components, and storage [39].

The cloud-computing paradigm is used to handle IoT challenges mainly related to storage and networking [4]. Cloud applications generalize machine-learning models by training cloud models using data that were collected from IoT devices located in several environments [4]. The cloud-computing paradigm partially addresses IoT issues [5]. Following are the advantages of the cloud-computing paradigm for large-scale deployments for IoT networks (LSD-IoT):

- Storage: cloud computing provides low-cost secure storage and computing services for IoT data [40], and provides customers with the ability to access their data anytime and anywhere and pay only for what they store.
- Scalability: cloud computing can handle the expanding number of IoT devices by troubleshooting and efficiently maintaining them. Resources, such as bandwidth and storage, could also be optimized according to IoT application demands [41,42,43].
- Performance: cloud computing could enhance the performance by processing IoT data in the cloud and reduce the burden on IoT devices, in addition to the high processing resources of cloud computing better than IoT devices [17].

Although the IoT is largely dependent on cloud architectures, cloud computing cannot always make optimal decisions regarding many aspects such as processing and data storage. Storing every single reading by a temperature sensor is inefficient. Thus, a new architecture needs to be considered, due to the following reasons:

- Latency: many IoT applications interacted with the real environment through sensing and actuation, which makes these applications latency-critical applications, such as e-health applications. Integrating IoT applications with cloud computing alone is not enough due to the distance of cloud computing resources of IoT devices, therefore, integrating cloud with edge computing solves this issue as discussed in the next subsection [44,45].
- Data size: the increasing number of IoT devices means more bandwidth needed to transmit IoT data to the cloud especially when smartphones are capable to send streaming videos and photos to the cloud, which leads to bringing cloud-like services near to the end-user to reduce the bandwidth required to transmit IoT data [46].
- Constrained resources: IoT devices e.g sensors are constrained with resources such as a limited battery. Transmitting IoT data to cloud servers consumes battery; therefore, bringing cloud services near to IoT devices is a requirement for resource-constrained devices to ensure the quality of service in IoT networks [47].
- Availability: IoT devices must be connected to the cloud, especially in time-critical applications, and to obtain this condition IoT devices should be always connected to the internet, which is a burden on IoT devices, especially with constrained resources [48].

### 3.3. Edge Computing

This section illustrates the definition of edge computing, edge-computing implementation, and edge intelligence.

#### 3.3.1. Definition of Edge Computing

Edge computing is a new distributed IT architecture, in which data-storage, services, and computing applications are partially or fully pushed from centralized nodes to near the end-user. IoT devices generate tremendous amounts of data; generated data could reach 500 zettabytes [19]. This leads to issues in bandwidth, storage, and processing. Edge-computing technology helps in real-time applications by reducing the latency and response time [19]. Edge computing extends cloud-computing capabilities to the edge of the network. Edge and cloud complement each other; edge computing ensures the continuity of the services, and the cloud manages the network. Storage, control, and communication are distributed near the end-user by the edge device. Thus, employing edge computing in the network enhances the network in different aspects:

- Efficiency: an edge device takes full advantage of the available resources by allocating storage, computing, and control functions to available resources in any place between the end-user and cloud [19]. This allows for IoT devices to efficiently utilize the shared edge-computing resources.
- Cognition: an edge device is conscious of customer requirements [49]. For example, in an e-health system, especially in emergency patient situations, the physical health of patients is monitored via IoT devices, and computation-resource allocation is adjusted based on users’ health-risk grade [50].
- Agility: it is quicker and inexpensive to experiment with edge devices and clients, because data processing and storage are done close to the end user [51].
- Latency: edge computing supports time-critical applications by enabling data analysis and data processing near the end-user, which grants IoT applications the ability to make decisions faster and better [52].

#### 3.3.2. Edge-Computing Implementation

Edge computing has three implementation forms: cloudlet, mobile edge computing (MEC), and fog computing [53]. These differ in terms of functionality, architecture, and node location. This subsection illustrates the differences between these implementations.

- **Cloudlet** is a group of computers that represent a small data center referring to cloudlet nodes dedicated to providing services to IoT devices located within the same geographical area [54].
- **Fog computing** was first introduced by Bonomi et al. [55] in 2012. Fog terminology comes from the fact that fog is closer to the end-user than to the cloud [56]. Fog computing is a decentralized infrastructure of computing nodes in which the services provided to end-users are located between end-users and the cloud [57]. Fog-computing nodes are heterogeneous; thus, various types of devices could be fog-computing devices: switches [58], industrial controllers [59], and access points [60]. This leads to the flexibility of fog computing because fog-computing nodes could be located anywhere between end-users and the cloud [61]. Fog-computing nodes also transfer small payloads faster than cloudlets do. However, it is four times slower than cloudlet is when transferring larger payloads [62].
- **Mobile edge computing** (MEC) or multiaccess edge computing is a network that provides cloud-computing services to mobile devices at the edge of a mobile network to reduce latency [63,64]. On the other hand, MEC enhances cellular network services with low latency [65] and high bandwidth [66] analyzes huge amounts of data before sending them to the cloud [67], and provides context-aware services [68]. Unlike fog-computing nodes, MEC servers could be deployed at a 3G radio controller or an LTE macro base station [69].

#### 3.3.3. Edge Intelligence

Conventional edge-computing devices are of low intelligence capabilities. These devices are responsible for local data processing, such as feature extraction [24] and transmitting data to cloud servers. However, when equipping edge devices with machine-learning capabilities, edge devices become more intelligent by increasing their ability to analyze data and make decisions without the need to connect to the cloud [70]. This reduces the latency time and time-to-action [71]. The demand for moving the intelligence from the cloud to edge device is an attractive research area due to different reasons:

- Security: when IoT data are transmitted to cloud servers for analysis, this can cause security and privacy issues when using public and private infrastructures. Processing data near the end-user protects user privacy [72].
- Performance: in time-critical IoT applications, such as in vehicular communications, any small delay in latency is noticeable [73]; therefore, edge processing is desirable.
- Bandwidth: the increasing number of devices that continuously generate data consumes bandwidth, such as cameras that keep sending videos and photos [35]. Processing data at the edge reduces the Internet bandwidth consumption.
- Data integrity: transmitting data to edge devices does not require compression or modifying the data format. Data are also not exposed to noise during the transmission process [35].

Applying machine learning (ML) algorithms to edge-computing devices has several challenges:

- Complexity: ML algorithms usually run on powerful devices with good resources such as computing power and memory. On the other hand, edge-computing devices may vary in terms of resources. Thus, low-complexity ML techniques are required [74].
- Memory constraints: artificial-intelligence (AI) techniques, such as neural networks, require much memory space to save the parameters of the AI model and the weight vectors that describe the classification model. Therefore, there is a need to design AI techniques that can run on resource-constrained devices [74].

An example of machine-learning techniques that are used in edge computing is deep learning (DL). DL techniques are multilayer neural networks, with each layer being responsible for extracting specific features from the input dataset. Deep learning is appropriate for edge computing since it is possible to offload parts of learning layers in edge devices and transfer the reduced intermediate data to the centralized cloud server. Deep learning could also automatically extract features for different applications [75]. However, deploying DL in edge infrastructure has several challenges, including:

1. Most deep-learning techniques need cloud assistance [75].
2. Edge-computing nodes are distributed over the network, and each edge device may have specific analytic capabilities; therefore, a service-discovery approach is needed in order to identify an appropriate edge device [76].
3. Distributing streaming data to ideal edge devices and dividing tasks among them requires special algorithms and careful considerations [76].

### 3.4. Large-Scale IoT Deployments

**Figure 2:**
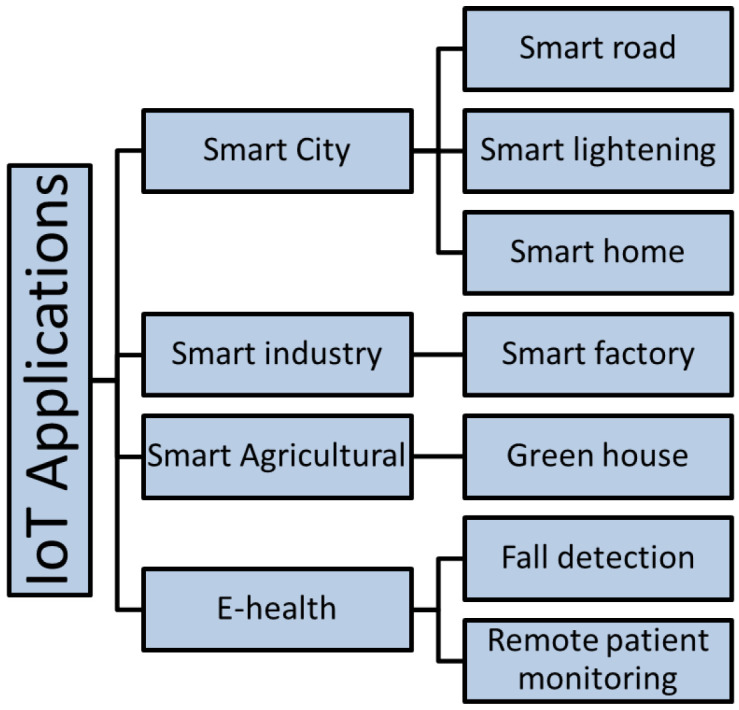
Internet of Things (IoT) applications.

IoT deployments can be of small [77,78] or large scale [79,80,81]. IoT-LSDs are generally characterized by heterogeneity [82] and large-scale IoT data. Increasing the number of connected IoT devices increases the data size that they generated. Shi [19] reported that generated data could reach 500 zettabytes. Moreover, transit traffic is expected to reach 10.9 zettabytes. Such characteristics can, in turn, lead to challenges that are not directly solvable while using conventional methods. Cloud-assisted techniques are also not adequate to handle these challenges, especially with time-critical applications. Moving IoT data from IoT devices to data centers located in the cloud increases the delay and communication overhead. Figure 2 shows possible areas of this deployment.

#### 3.4.1. Smart Cities

A smart or intelligent city is one that uses various types of IoT devices to collect data and uses these data to manage events and deliver insights. The following are examples of IoT applications in smart cities:

- Smart homes: controls energy and water consumption, home applications remotely, and acts as a security system [83].
- Smart lighting: controls lighting to support energy consumption, adjusting lights based on city conditions [84].
- Smart roads: enhances the safety of drivers, traffic management [85], and energy efficiency [86]. This helps drivers to find free parking and park their cars in order to reduce road congestion using wireless parking sensors [87].

#### 3.4.2. Smart Industry

Using IoT devices in industries enhances automation, predictive maintenance, and monitoring events remotely [88]. A smart factory is highly digitized and equipped with sensors that communicate with each other in order to improve processes via automation, self-enhancement, and optimization.

#### 3.4.3. Smart Agriculture

IoT enhances agriculture in several domains. For example, in agricultural applications, greenhouse monitoring enhances planting in greenhouses and protects plants from many diseases through sensors that monitor soil, structures, and heat [14].

#### 3.4.4. E-Health

IoT devices can collect health data, transform them into information, and use them to enhance the quality of health services. The following are some e-health IoT applications:

- Fall detection: designed to more effectively detect falls for the elderly [89,90,91].
- Remote patient monitoring: uses wearable sensors to remotely monitor patients [92,93].

## 4. Related Work

This section reviews the literature of related surveys done in the areas of IoT, IoT architectures, and edge/fog computing. There are 19 key surveys that are available between 2010 and 2020, and this survey compares them in this section.

Lin et al. [94] provided a comprehensive review of IoT including enabling technologies, IoT architectures, and privacy and security issues. They also surveyed integrating IoT with fog/edge computing in terms of applications. However, they did not survey IoT applications. Al-Fuqaha et al. in [21] provide a comprehensive overview of IoT enabling technologies, applications, and protocols. Maier [95] compared IoT applications based on popularity and consumer type according to the following classes: personal, smart environments, homes, and vehicles. Pflanzner et al. [96] studied 20 IoT use cases from three main IoT applications: smart regions, smart cities, and smart homes. They categorized IoT applications based on several categories: (1) context based on fields and type of IoT application; (2) participants: focuses on application size, i.e., the number of users and of devices; (3) sensors: focuses on required sensor types in the IoT application; (4) network requirements such as network speed; and (5) other: for example, security requirements of the application. Atzori et al. [97] addressed the vision of the IoT paradigm, surveyed IoT-enabling technologies, and studied the potential IoT applications. Asghari et al. [98] classified IoT applications based on solutions that aim to support challenges in the IoT domain. The survey in [99] studied IoT architectures for domain-specific applications, and briefly summarized IoT cloud systems that support data analysis. In [100], Razzarue et al. surveyed IoT middleware systems against IoT requirements, including architectural requirements. Lao et al. in [101] surveyed IoT applications for blockchain systems. Farahzadi et al. [102] presented cloud-of-things middleware and studied various architectural styles of middleware and service domains. Sethi et al. [103] proposed a taxonomy for IoT technology, studied the architectures of IoT in terms of three-layer, five-layer, fog-, and cloud-based architectures, and social IoT that focuses on the relationship between IoT objects and applications. Abbas et al. [104] surveyed IoT applications in terms of emerging scenarios, and studied open research challenges for IoT paradigms. Miorandi et al. [105] studied the IoT in terms of concept and vision, applications, technologies, and research challenges. Dastjerdi et al. [106] surveyed IoT applications that benefit from fog computing, and then proposed a reference architecture for IoT, of which the paradigm is based on a software-defined network (SDN) and fog computing. In [107], Mahmud et al. proposed a taxonomy for the challenges of fog computing. Markus et al. [108] proposed a taxonomy of fog-computing simulation-environment modeling. Mouradian et al. [109] surveyed fog-computing architectures in terms of application-specific architectures and end-user-agnostic applications. They also surveyed fog-computing algorithms in terms of algorithms for computing, content storage, and distribution, end-user-specific applications, and energy consumption. They also studied fog-computing challenges and research directions. Atlam et al. [110] surveyed related works that integrate IoT with fog computing, presented IoT applications that benefit from fog computing, and studied fog challenges for IoT applications. Bellavista et al. [111] studied IoT application requirements that have to be accomplished by fog computing, proposed a fog conceptual architecture for IoT that is derived from IoT requirements, and proposed a taxonomy for components and used characteristics of fog-computing applications for IoT that is derived from the proposed conceptual architecture. Puliafito et al. [112] surveyed IoT applications that benefit from fog computing, studied research challenges for fog computing for IoT, and surveyed existing fog-computing platforms for IoT. Table 1 summarizes these surveys according to the dimensions of focus addressed by each survey. Dimensions include IoT architectures (IoT arch), IoT applications (IoT apps), IoT challenges, IoT technologies (IoT tech), fog-computing challenges (FC chall), fog-computing applications for IoT (FC-IoT apps), edge-computing architectures for IoT ECA-IoT, fog-computing challenges for IoT (FC-IoT chall), fog-computing algorithms (FC algo), and fog-computing platforms for IoT (FC-IoT platforms).This table shows that the majority of surveys focus on IoT applications, IoT technologies, and fog-computing applications for IoT; only one survey focuses on fog-computing challenges. To the best of our knowledge, we are the first who surveyed an ECA-IoT on the basis of IoT challenges. Additionally, most of these surveys focus on one or a few dimensions and do not provide a comprehensive review of ECAs-IoT while our survey paper does. By the same token, the surveys, in general, addressed the dimensions in a standalone aspect, rather than talking about the complete picture. Talking about IoT applications independently, or IoT challenges independently without considering edge computing, does not provide an accurate evaluation when looking at it from ECA-IoT perspective. Likewise, surveys focused on fog computing without considering IoT will be missing crucial evaluation elements.

## 5. Classifications of ECAs-IoT

**Figure 3:**
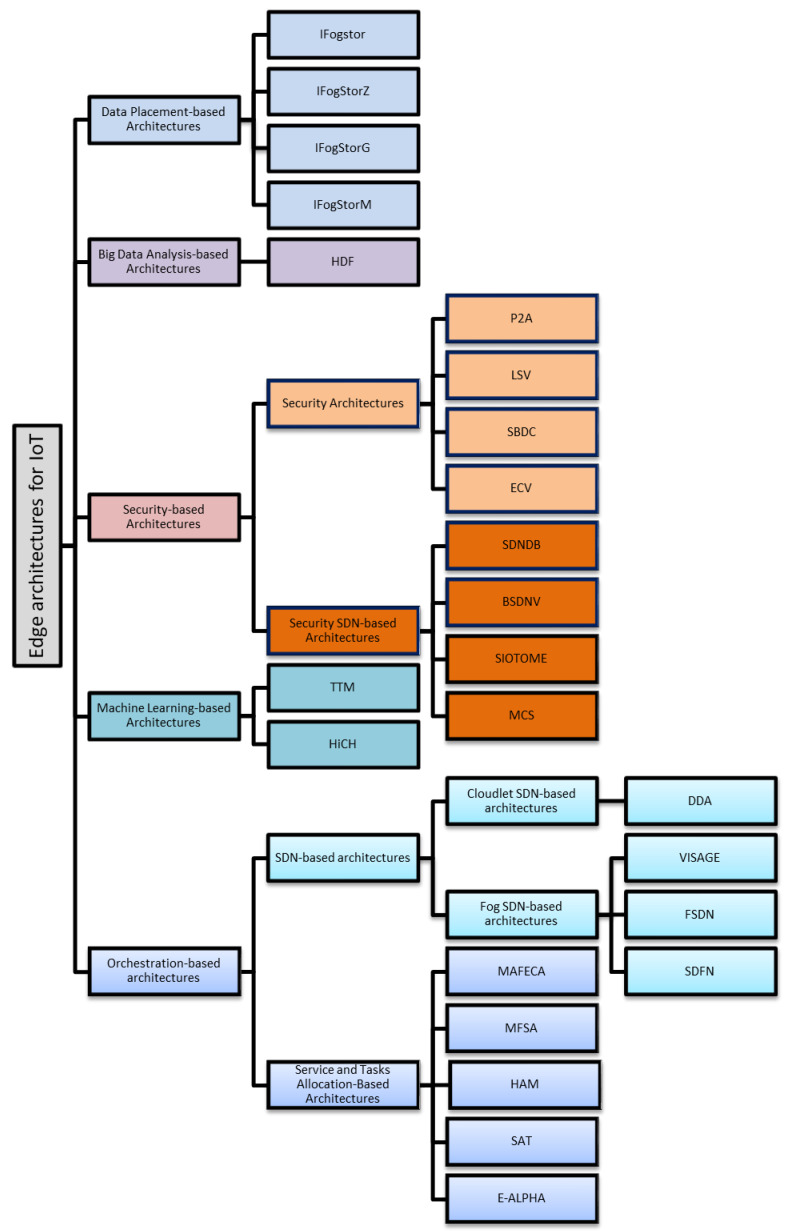
Edge-computing architectures (ECAs)-IoT taxonomy.

In this section, ECAs-IoT are classified according to the issues that they address for IoT applications. Current taxonomies do not consider ECAs-IoT besides they did not take challenges that face IoT networks in their taxonomies such as IoT data placement challenges, handling security challenges in ECAs-IoT architectures, handling big data analysis, etc., i.e linking IoT challenges with ECAs-IoT and, to the best of our knowledge, this taxonomy is the first taxonomy that links ECAs-IoT with IoT challenges in which this is an important research area, since Edge computing for IoT is being studied for a while. The taxonomy is summarized in Figure 3:

### 5.1. Data-Placement-Based Architectures to Reduce Latency

IoT networks generate a huge amount of data [113]. Critical IoT applications, such as e-health applications [114] require retrieving data with low latency [115,116]. ECAs-IoT face issues in placing IoT data in the appropriate edge nodes. The following are architectures proposed so far:

#### 5.1.1. IFogStor and IFogStorZ

The authors of [117] proposed IFogStor and IFogStorZ, taking advantage of fog-node variations and distributions to minimize the total latency of storing and retrieving IoT data in fog nodes. The system architecture components are a group of IoT devices, a group of fog nodes, a group of data centers, and a group of IoT services.

**Figure 4:**
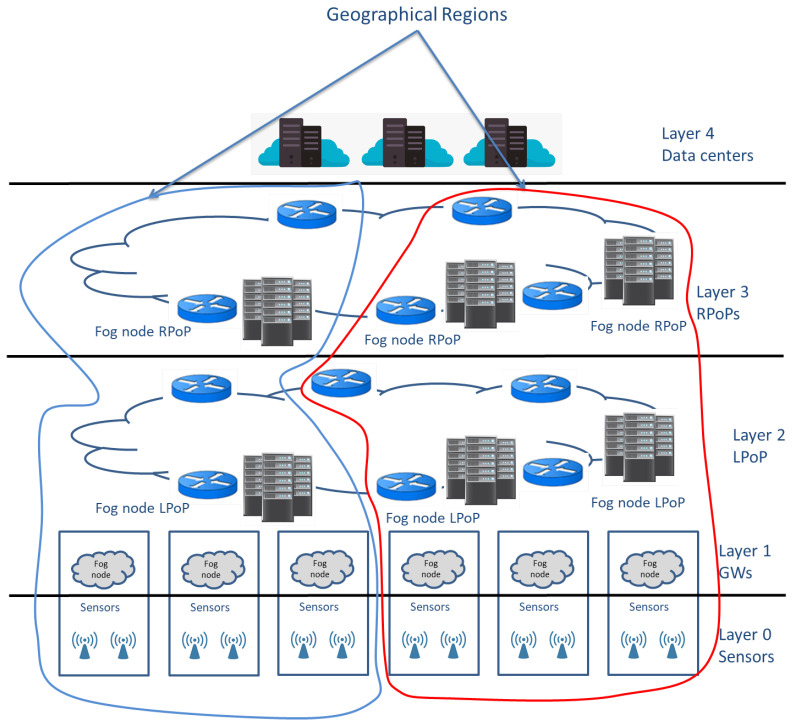
IFogStor system architecture.

IFogStor system architecture aims to reduce the overall network latency by finding efficient fog nodes to store and retrieve generated data. The data are placed in a robust node for run-time execution. This node knows information about data flow, the latency of the existing network, free storage capacity, and applications instance places. IFogStor system architecture consists of three main classes of actors shown in Figure 4: (1) data hosts, specialized nodes that store IoT data, which could be a fog node or a data center. They can exist in any layer excluding layer 0, as shown in Figure 4. (2) Data producers, which are nodes that generate data. This type of node can exist in different layers. (3) Data consumers, which are nodes that process or read IoT data, and they can exist in different layers. Fog nodes could play the role of data host, producer, and consumer at the same time due to their abilities.

Two solutions were proposed to solve the problem:

- IFogStor: an exact approach that solves the problem of data placement like a single integer program. It finds the optimal placement for small-scale applications; however, for large-scale applications, its performance is unacceptable.
- IFogStorZ: a divide-and-conquer heuristic approach that divides geographical locations using regional points of presence (RPoPs) as points of partitioning. Each location is a subproblem, and solutions are then aggregated in order to provide a global solution. However, this solution does not find the optimal placement, but it drastically improves data latency.

#### 5.1.2. IFogStorG

Although IFogStorZ is simple to implement, a considerable loss of optimality occurs when data producers are far from data consumers. Besides, IoT services and the number of fog nodes may vary among subregions. Subsequently, unbalanced subproblems are found. The aim of IFogStorG [118] is to enhance runtime performance and minimize the complexity of the data-placement strategy. Thus, enhanced technology can handle dynamic changes in the network topology. In the partitioning step, the focus is on the following constraints: (1) the numbers of data items and data hosts are balanced among subregions to enhance the complexity and (2) data consumers and data producers are maintained as much as possible in the same subregion, since the efficiency of the strategy depends on the location relationship among them. As in IFogStor, the authors used matrices to map data consumers and data producers, fog nodes with their executed data producer, and fog nodes with implemented data consumers, and they used an adjacency matrix to represent the latency values existing in the infrastructure. Thereafter, they solved for each subgraph while using the IFogStor strategy. Subsequently, the results for each subgraph are aggregated, and the final global solution is found. They evaluated their strategy by using a smart city as a use case. However, if the number of data subscribers increases, then the data replica and network overhead also increase; therefore, a trade-off between latency minimization and the number of data replicas should be made.

#### 5.1.3. Multireplica Data-Placement Strategy (IFogStorM)

Huang et al. [119] handled the latency issue that is generated when data consumers are located in different geographical areas that are subscribing to the same data, while only one replica of these data exists in the proper fog node. To solve this issue, they proposed a greedy algorithm, called IFogStorM, to minimize overall latency. Results showed that overall latency was reduced by 10% more than in IFogStorG [118] and by 6% more than in IFogStorZ [117].

Table 2 shows the differences among the data-placement architectures in terms of techniques, improvements, and weaknesses. The table shows that existing data-placement architectures use several techniques to handle the data placement issue and minimize the latency while accessing the data. Several techniques were used such as divide and concur and graph partitioning. Furthermore, some techniques do not scale well and produce a poor performance when used in an LSD-IoT environment.

### 5.2. Orchestration-Based ECAs-IoT

Managing IoT networks is considered to be an important challenge because it enhances security and system reliability, and simplifies network maintenance [120,121]. Some ECAs-IoT manage IoT networks by employing a software-defined network as the core solution, while other ECAs-IoT employ other techniques.

#### 5.2.1. Service- and Task-Allocation-Based Architectures

Edge computing enhances IoT networks by managing sensitive service processing and task allocation to appropriate edge nodes. This section discusses ECAs-IoT that handle task and service allocation in IoT networks.

##### Mobile Fog Service Allocation (MFSA)

Edge computing enhances IoT networks by managing sensitive service processing and task allocation to appropriate edge nodes. In MFSA, Daneshfar et al. [122] minimized the overall cost of providing services by using integer-programming (IP) formulation, while allocating requests to available resources. Each server has a “probability of availability” in the formulation. A middleware controller exists between users and fog nodes that has the correct information about the entire architecture. It also knows a nondeterministic availability component of each server. To handle this component, each user multicasts its service request to several servers. However, the number of servers that the user can send requests to is bounded. They also proposed that sending a request to each server has a cost, and each user has a certain budget. Each server resources could be used by multiple users if and only if the overall provided services from the server do not exceed its capacity. The probability of servers’ availabilities is independent. Each service has a cost. Additionally, they determined the QoS level by using a set of constraints. Constraints include the probability of availability for the server targeted by the user. The aim of solving this problem is to reduce the total cost of allocating services.

##### Multiagent-Based Flexible ECA-IoT (MAFECA)

Another task-assignment ECA-IoT is multiagent-based flexible ECA-IoT (MAFECA). In [123], task assignment between edge devices and the cloud is optimized by proposing a flexible architecture that handles the traditional IoT network challenges. In this architecture, two system abilities are adopted: user-orientation and environment-adaptation abilities. User-orientation ability uses that were collected information from IoT devices such as user behavior to afford services for each user in real-time. Environment adaptation determines the processing location, whether it is in an edge device or the cloud, depending on the quantity and the quality of the task.

##### Hierarchical Architecture to Place Mobile Workloads (HAM)

Tong et al. [124] proposed a hierarchical architecture to place mobile workloads (HAM), a hierarchical architecture to handle service allocation to mobile workloads, by proposing a placement algorithm that places mobile workloads between several layers and decides how much computation capacity each workload requires.

##### Scalable IoT Architecture Based on Transparent Computing (SAT)

Ren et. al [125] proposed an architecture that enhances service allocation by leveraging transparent computing that can fully take advantage of edge-computing devices to enhance scalability and reduce response delay. This architecture consists of several layers: end-user layer consisting of IoT devices; **end server layer**, responsible for distributing services to end-users; core network layer, responsible for communication between edge-computing devices and the cloud; cloud layer, which consists of powerful computing and storage resources to handle large-scale and complex data; and, management and interface layer, which is responsible for managing the entire architecture.

##### Edge-Based Assisted Living Platform for Home Care (E-ALPHA)

Aloi et al. [126] proposed an architecture that enhances e-health applications and consists of the following components: manager, responsible for coordinating other components in the architecture; communication engine, responsible for managing radio interfaces; device handler, responsible for separating the technology-based operations of communication-based services; device controller, responsible for dynamically loading specific protocols; database, which stores devices status; interface management, which supports communication between E-ALPHA devices and cloud; and GUI, which provides a way to access software settings modules. This architecture was simulated while using EdgeCloudSim simulator [127].

#### 5.2.2. SDN-Based Fog Architectures

Resources, data, and services should be managed in IoT networks [128]. Software-defined networks (SDN) could enhance ECAs-IoT. This section discusses ECAs-IoT that apply SDN technology to orchestrate the network:

##### Multilevel SDN-Based 5G Vehicular Architecture with Vehicles as Fog Computing Infrastructures (VISAGE)

**Figure 5:**
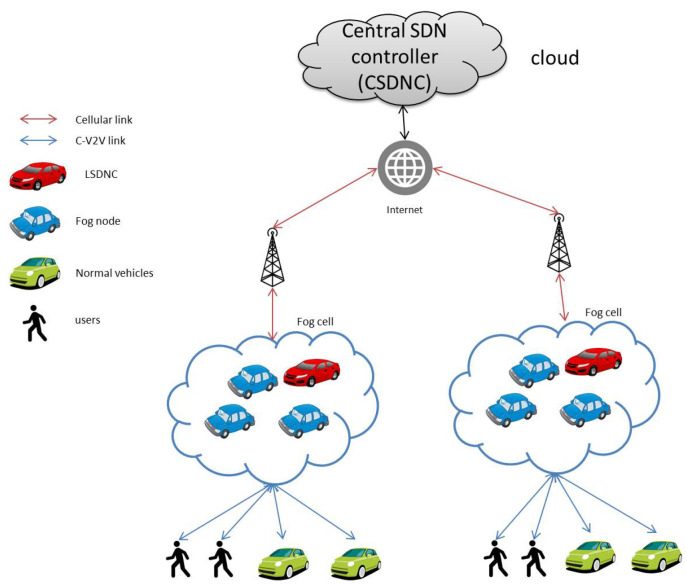
VISAGE architecture.

The number of vehicles on roads continue to increase. Therefore, Soua et al. in [129] proposed a model for future 5G technology for a vehicular ad hoc networks (5G-VANETS) system; the system contains two submodels: the central SDN controller (CSDNC) and local SDN controller (LSDNC). This takes advantage of using vehicles as fog nodes, and even parked vehicles could be used as fog nodes. The architecture in [129] contains the following components (as shown in Figure 5): CSDNC is permanent and resides in the cloud; this part represents global intelligence and orchestrates the whole system. LSDNC centralizes intelligence and represents local intelligence. It is controlled by the CSDNC, and it controls the local fog cell. Fog nodes form the fog cell and are controlled by the LSDNC. Customers cannot compute their tasks, and fog nodes provide them with this service. They could be vehicles, individuals, or organizations. Base stations maintain the connectivity between cloud and fog nodes. In VISAGE, the LSDNC multicasts its Fog-SDN capabilities. Each vehicle could participate as a fog node or take a service from the fog cell. The fog cells could be connected to the Internet through the LSDNC. Thereafter, the LSDNC communicates with the CSDNC, and the latter then orchestrates the resources.

##### SDN-Based VANET Architecture (FSDN)

The architecture in [130] enhances resources management in VANET. This architecture consists of the following components: SDN controller that resides in the cloud and is responsible for global intelligence; road-side unit controller (RSUC), responsible for controlling a group of road-side units (RSUs), forwarding data, storing information about local road systems, and performing timely services; RSU, responsible for forwarding data and controlled by the SDN controller; cellular base station, responsible for local intelligence, data forwarding, and delivering fog services. No performance evaluation is conducted for this architecture; however, this architecture lacks resource management and network orchestration.

##### Software Defined Fog-Computing Network Architectures for IoT (SDFN)

Tomovic et al. [131] proposed an integrated system that consists of SDN and fog computing; this architecture differs from previous architectures in that it is generic. Architecture components are: end devices, which are IoT devices; SDN controllers, responsible for orchestrating fog devices, selecting optimal access points of IoT devices, and having information about the network such as fog-device capabilities to assign tasks to them; fog infrastructure, consists of various types of devices and deployed hierarchically. Fog devices use application programming interfaces (APIs) to provide their services; cloud computing, located in the core of the network. This architecture adopts a hierarchical deployment where the same application could be run in multiple fog devices. Each fog device is responsible for running a specific task based on device capabilities. This architecture could be deployed in several IoT applications, such as inter-transportation systems, video surveillance, and precision agriculture. No simulation is performed in order to evaluate the architecture and there is no centralized control of the network.

#### 5.2.3. SDN-Based Cloudlet Architectures

This subsection illustrates architectures that employ an SDN and cloudlet to manage an IoT network, as follows:

##### Dynamic Distribution of IoT Analysis (DDA)

Munoz et al. [132] proposed an SDN-based multilayer architecture that monitors IoT flows, integrates techniques to avoid IoT traffic congestion, and distributes IoT data analysis between a data center (DC) and the edge of the network. This architecture is as follows: At the infrastructure layer, connectivity to DCs located at the edge of the network is provided, and the average bandwidth of the IoT flow is monitored. At the control layer, each DC located at the infrastructure layer has a dedicated DC controller. At the top of the DC controller, they also deploy a cloud orchestrator that provides federated cloud services. Each transport domain has an SDN controller; at the top of these controllers, there is an IoT-aware transport SDN orchestrator (TSDNO) that acts as a controller of controllers. TSDNO is also responsible for preventing IoT traffic congestion. At the top of cloud orchestrator, an IoT-aware global service orchestrator (GSO) is responsible for orchestrating global end-to-end services.

Table 3 shows the differences among management-based architectures in terms of techniques, factors of enhancement, and the weaknesses of each architecture. It shows that the goal of these architectures is to reduce latency and enhance scalability; it also shows that most orchestration-based architectures employ SDN in order to orchestrate the network. In general, management-based ECAs-IoT lack the support of one or more of the following: heterogeneity, LSD-IoT, simplicity. The heterogeneity is addressed well in IoT as a standalone concept, but it requires further consideration when introducing edge computing as part of the infrastructure. Likewise, the management of LSD-IoT requires additional levels of consideration, such as the participation of the Internet Service Providers (ISPs) in the edge network, the distribution of IoT devices over larger geographical areas, and multiple edge computing areas. Finally, keeping all of this simple, dynamic, and easy to manage is another challenge.

### 5.3. Big-Data-Analysis-Based Architectures

Sensors generate tremendous amounts of data. This subsection covers fog-computing architectures that are proposed to solve big-data-analysis issues.

#### Hierarchical Distributed Fog Computing Platform for Smart Cities (HDF)

**Figure 6:**
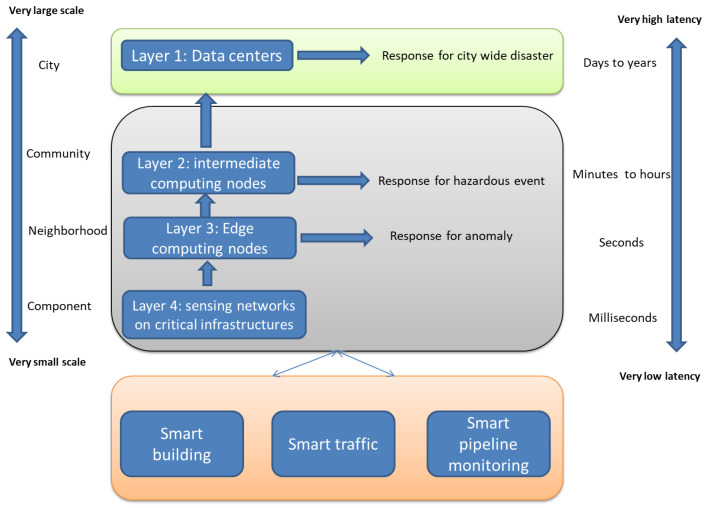
Hierarchical distributed fog computing.

Tang et al. [133] proposed a hierarchical architecture that consists of four tiers. Figure 6 shows the tiers of the proposed architecture. Layer 4 is the sensing network that contains various types of sensors that are distributed to different places in order to collect and generate data. Thereafter, raw data generated from the sensors are forwarded to Layer 3. Layer 3 consists of edge devices that are responsible for a group of sensors from Layer 4 that cover a small community or neighborhood. Edge devices in this layer provide timely data analysis. The output of those edge devices is separated into two parts: reports that are the results of analyzing the data, and feedback to the infrastructure to respond to threats that were monitored by sensors. Afterward, the edges in Layer 3 are grouped, and each group is connected to one of the intermediate computing nodes in Layer 2. This node connects temporal and spatial data to recognize possible dangerous events, and it can quickly control a dangerous situation. The last layer, which is the cloud-computing data center, provides overall infrastructure analysis, monitoring, and controlling, such as long-term pattern recognition. In order to evaluate the infrastructure, they built a prototype for a pipeline system and simulated 12 different events around the sensors. In order to recognize the events, they trained a hidden Markov model. The results showed that using fog computing with cloud resources reduces latency with big-data analysis.

In [134,135], a similar architecture was suggested while using a hierarchical edge- and cloud-computing model to collect data from several sensors and sources. Several sensors push data to the first level of collectors, an edge level that gets pushed to a general cloud provider. The general provider has all data fused into one place. The data can then be retrieved by customized providers that can offer further information derived from the fused data where big-data analysis can be applied.

### 5.4. Security-Based Architectures

The nature of IoT networks leads to various security threats, such as the confidentiality of user data and data authentication. Edge-computing technology solves some of IoT networks security threats, this section discusses ECAs-IoT that handles security issued on IoT networks:

#### 5.4.1. Security Architecture

This subsection illustrates architectures that handle security issues in IoT networks and do not employ SDNs in their architectures, as follows:

##### Privacy Preservation While Aggregating the Data (P2A)

**Figure 7:**
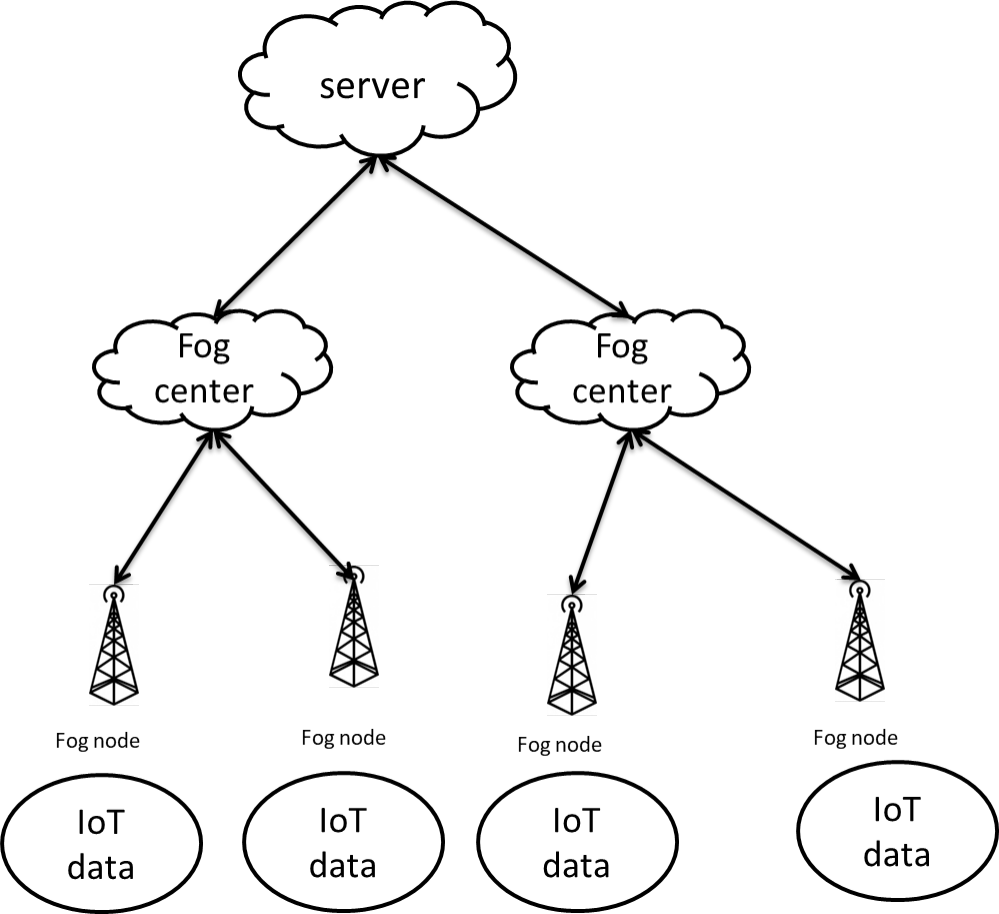
Privacy-preserving architecture.

Yang et al. [136] proposed architecture in order to preserve sensor data privacy that handles multifunctional aggregation, communication overhead, and computation overhead. This architecture consists of four main entities, as shown in Figure 7: sensors, fog nodes, fog centers, and a cloud server. Sensors are located in smart devices to collect data. In order to preserve privacy, the collected data are divided into two parts and sent to two different fog nodes. The fog nodes serve as storage nodes to help in aggregating data when aggregation queries come from fog centers. Thereafter, fog centers collect the results of queries that come from the fog nodes. Subsequently, the main query results are calculated and sent to the cloud center. The cloud center works as an aggregation application and it is managed by a service provider.

In their architecture, fog centers and cloud centers are untrusted entities, as they try to collect the original data that are confidential. Fog nodes are semi-trusted, so they have a curiosity about the original data, as they cannot collude with each other.

In order to aggregate data and preserve privacy at the same time, Yang et al. [136] proposed a multifunctional aggregation framework that is based on machine learning. The model does not send the original data, it only sends a predicted value by training the model in order to predict various query results. The training data are the collected data from each region.

The following presents how the protocol works. The cloud center sends queries to fog centers; permissible queries that the cloud center could send are average, q-percentile, min, max, summation aggregation, and medium. The cloud center sends all of these queries to the fog center. However, the fog center is unable to answer cloud-center queries; therefore, the fog center generates a set of queries on the basis of the original queries that came from the cloud center.

The fog-node sensory data are reported from the sensors after dividing sensory data into two parts. The received data are trained and predicted on the basis of the new set of queries that were generated by the fog center. Lastly, the fog center receives the predicted values and re-sends them to the cloud center.

##### Lightweight Security Architecture Based on Embedded Virtualization and Trust Mechanisms for IoT Edge Devices (LSV)

The authors in [137] proposed a secure ECA-IoT in order to secure edge devices without the need to re-engineer the applications installed in edge devices by integrating embedded virtualization with trust mechanisms. The proposed architecture ensures some security requirements: the confidentiality of permanently stored elements, executed-code authenticity, and run-time state integrity. The security architecture consists of four security mechanisms: security by separation, secure boot, secure key storage, and secure interdomain communication.

The edge device is secured on the basis of two factors: (1) root of trust (RoT), in which the edge device is unclonable in addition to the integrity, nonrepudiation, and authenticity of the running software at edge devices; and (2) chain of trust (CoT), in which the edge device is designed to boot up only if cryptographically signed software by a trusted entity is first executed using public-key cryptography. In addition, the keys are stored in specialized secure hardware; this hardware is also responsible for verification and RoT processes. As a result, this architecture of embedding virtualization consists of a number of virtual machines (VM) with different vendors, performing another level of secure boot verification.

The system architecture is still vulnerable to run-time attacks, even if the CoT is established, and using hardware-assisted virtualization maintains a trusted execution environment (TEE); thus, securing it through run time is required. The system architecture was evaluated on the basis of three metrics: memory footprint, performance, and inter-VM communications latency. The results showed that edge-device protection could be achieved without the need to re-engineer applications at the edge.

##### A Secure IoT Service Architecture with Efficient Balance Dynamics Based on Cloud and Edge Computing (SBDC)

Conventional security mechanisms cannot resist IoT attacks due to limitations in IoT devices. Wang et al. [138] established a secure architecture on the basis of trust mechanisms and service templates in order to resist such attacks and repeated handles or similar service requests by leveraging the edge devices that were distributed over the network. The service template consists of two templates: the service and service-parsing templates.

This architecture is based on three basic components: (1) the edge network, (2) the edge platform, and (3) the cloud. These components are distributed over three layers, (1) data collection, (2) data processing, and (3) app-service layers. The edge network is in the data-collection layer, the edge platform is in the data-processing and application-service layers, and the cloud is located in the app-service layer. This architecture has the following advantages: (1) it creates a trust state of IoT devices and chooses a trusted IoT device to perform services, (2) dynamically adjusts IoT load, and (3) serves end-users’ requirements such as integrity and precision. This edge platform is responsible for the following tasks: performing virtualization processes through converting physical devices into virtual devices; dynamically adjusting cloud load by performing some services on the edge layer; ensuring the IoT reliability by cooperating with the edge network at the data level, and establishing the service-parsing template that is responsible for storing the matching information and parsing strategies.

The cloud is responsible for creating and storing service-parameter templates, and storing matching information, performing parsing processes in order to find matching services in the cloud, processing services that require more resources than services existing in the edge-processing layer do, logging old data in order to be used for further analysis, and data mining.

Extensive experiments were done while using the MATLAB platform in order to evaluate their architecture. The architecture consists of four IoT networks and one cloud. Each IoT network has one edge platform. The results show that this architecture can enhance the efficiency of services and data trustworthiness.

##### SIOTOME: Edge-ISP Collaborative Architecture for IoT Security

Haddadi et al. [139] performed cooperation between the edge of the network and the Internet service provider (ISP) for the early detection of threats and vulnerabilities produced from IoT devices. Unlike traditional networks in which the intrusion-detection system defends a single domain, SIOTOME learns from various domains to identify various attacks. Domains could be an individual ISP, cloud network, or an individual home network.

The SIOTOME system architecture consists of two high levels of domains: SIOTOME/edge and SIOTOME/cloud. The architecture was applied to a smart home; in the system architecture, the following components are found: the gateway that provides the home’s network with Internet connectivity with the ISP and forces the internal home network to be under one controller; the edge data collector that is responsible for collecting the IoT data; the edge analyzerthat is responsible for analyzing collected data and IoT device behavior; and, edge controller, which is based on SDN and is responsible for gateway configuration.

The SIOTOME/cloud component consists of the following components: the cloud collector, which is responsible for collecting reports from the edge data collector and monitoring whether the ISP network has malicious activity; the cloud analyzer, which is identical to the edge analyzer that analyzes data collected by the collector; the cloud controller, an SDN controller that countermeasures threats that are found by the analyzer and controls data at the ISP level; the cross-domain controller that controls traffic among domains; and, the secure communication component that maintains secure communication between different system components.

##### Edge-Computing Architecture for Mobile Crowd Sensing (MCS)

Marjanovic et al. [140] proposed an architecture to serve mobile crowd sensing that consists of four layers: user equipment layer, consisting of IoT devices such as wearable sensors; edge-computing layer, responsible for worker management in certain geographical areas; cloud-computing layer; responsible for processing complex data; and, application layer, responsible for data analysis. This architecture has several benefits, including protecting data privacy by partitioning data and distributing them to servers, and decreasing latency, because it sends a notification to mobile devices when a mobile crowd-sensing scenario occurs.

##### ECA-IoT Integrating Virtual IoT Devices (ECV)

Kanti et al. [141] proposed an ECA-IoT to enhance smart cities. This architecture provides an intermediate layer for IoT data processing. This architecture consists of six components: collection proxies, responsible for connecting each IoT device to other components in the architecture; data validation, which ensures the integrity of collected data; metadata annotation, where additional data are added to the original data after verifying their correctness in order to make data processing easier at virtual IoT devices; security, which performs symmetric data encryption for IoT data before delivering them to the cloud servers to preserve their privacy; virtual IoT Device (VID), responsible for IoT data processing, and assisting in data validation and annotation; and, actuation, responsible for triggering an actuation if certain conditions are met.

#### 5.4.2. Security SDN-Based Architectures

This subsection covers ECAs-IoT that handle security issues in IoT networks and employ SDN in their architectures.

##### Software-Defined Fog-Node-Based Distributed Blockchain Cloud Architecture (SDNDB)

Sharma et al. [142] proposed a fog architecture based on SDN and blockchain to enhance latency in a secure manner. The system architecture consists of three layers: Device layer: consists of IoT devices that sense and transmit data to the upper layer. Fog layer: consists of a blockchain-based SDN controller that represents fog nodes. Each node is responsible for a small associated community which is responsible for analyzing IoT data and providing services in a timely manner. Each fog node is responsible for securing the network against a saturation attack [143] by applying a packet-migration function and analysis function of the flow rule. Cloud layer: consists of a blockchain-based distributed cloud that is responsible for analyzing behaviors, recognizing long-term patterns, and detecting large-scale events. Clients are allowed to search for, use, and provide services. The performance of this architecture is evaluated by using incurred delay, response time, and the accuracy of detecting the saturation attack with a testbed. The results showed that this architecture functions better than a conventional core-based cloud-computing infrastructure does.

##### Blockchain-SDN-Enabled Internet-of-Vehicles Environment for Fog Computing and 5G Networks (BSDNV)

Gao et al. [144] proposed a blockchain-SDN architecture to enhance trust in networking platforms by integrating blockchain with SDN for VANET networks with 5G and fog computing. Blockchain establishes trust within system components, SDN ensures accomplishing control processes, and fog computing avoids frequent handovers. BSDNV components are Vehicles: these components consist of an on-board unit (OBU) that is connected to the SDN, acting as end-user. OBUs are responsible for packet forwarding and monitoring vehicle information such as vehicle speed and environmental information. RSUs: they are connected to the broadband unit (BBU). Base stations: t connected to the BBU. Fog nodes: controlled by the SDN controller. Fog zones: each zone represents a cluster of fog nodes. RSU hubs (RSUH): these components are responsible for controlling overhead among vehicles, connecting fog zones together and with the SDN controller, and reducing SDN controller overhead, because they make decisions on the basis of their local intelligence. SDN controller: this component is considered the core of this architecture, and it is responsible for allocating resources, generating rules, data preprocessing and analysis, mobility management, and generating rule. Blockchain: nodes in the blockchain consist of an access controller, policy-management server, data-management server, and an authentication server. The blockchain collaborates with the SDN in order to ensure the effectiveness and the efficiency of the network. Simulation results show that this architecture improves a VANET in terms of security and orchestration.

Table 4 presents ECAs-IoT that address partially one or more security aspect and compares among them in terms of deployed techniques, security issues addressed, and architecture’ weaknesses. The table shows the security issues addressed along with the techniques used to handle them, such as ML techniques. Most security ECAs-IOT focus on data privacy, and there are several security requirements that should be taken into consideration, such as integrity and availability. In addition, scalability is a common concern as handling security issues in an LSD-IoT environment requires avoiding static security solutions and proprietary security protocols.

### 5.5. Machine-Learning-Based Architectures

Applying machine-learning techniques in edge devices enhances IoT applications. This section discusses ECAs-IoT that apply ML techniques in edge devices:

#### 5.5.1. Hierarchical Fog-Assisted Computing Architecture (HiCH)

Conventional edge infrastructures apply traditional machine-learning techniques in the cloud, which leads to delays in the response time. Therefore, the authors of [145] proposed an HiCH architecture that is based on the MAPE-K model that contains four main components: manage, analyze, plan, and execute; each component is responsible for a role: Monitor: this component consists of an analog-to-digital converter that converts analog signals to digital form. In addition, it has a microcontroller that is responsible for aggregating data. Data are partitioned and packetized depending on the data type generated from sensors, and transmitted to the network management. Analyze: this component is in the cloud server. Data analysis is done by applying different machine-learning techniques on the basis of the generated data type from the sensors. The component generates a model from training a sensory dataset. This model is executed at the edge device in the plan component. However, the ML technique that is used to generate the model must fit the edge-device capabilities. The linear classifier most fits the HiCH architecture [146]. Plan: this component is similar to feature extraction. Features are extracted from test sensory data, and extracted features generate new input to the decision-making unit. A decision vector unit is generated and sent to the execute unit for system actuation. Execute: sends updates to the previous parts in the model and sets system behaviors during monitoring. System management: this part is responsible for data transmission.

#### 5.5.2. Transferring Trained Models (TTM)

**Figure 8:**
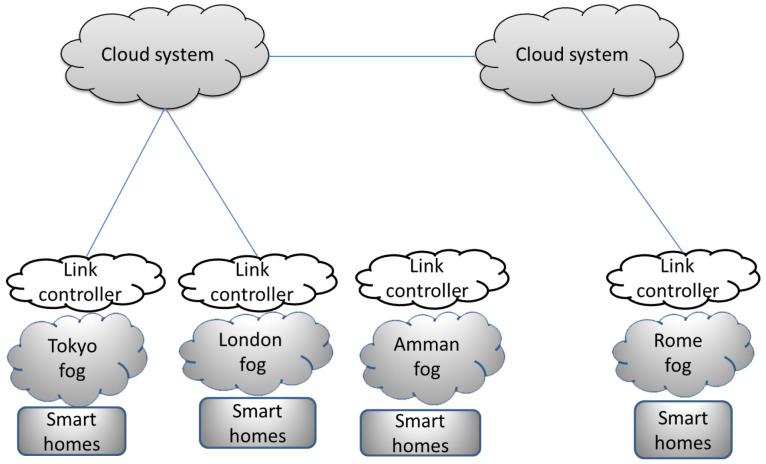
Transferring-model architecture.

In-home applications suffer from a lack of training data that are needed to train models to apply new services. Transferring trained models among various smart homes has become a major field of research. In [147], the authors proposed an architecture that has the ability to transfer activity-recognition models from a source home to target home. In their proposed architecture, three layers were defined, as shown in Figure 8. First, a set of smart homes that are grouped on the basis of their cities. Second, the fog-node layer where each fog node is responsible for a specific city. Third, the cloud-system layer that provides the ability to the fog nodes to communicate with each others and manage the shared settings, environmental parameters, data, and knowledge. In order to transfer the trained model from a source home to a target home, two conditions should exist: model accuracy and homogeneity between the target environment and source environment. The first step of their proposed framework is feature analysis, which is in charge of extending source datasets to address heterogeneity in feature spaces among various sources of data. However, the number of generated features is very large; therefore, dimensionality reduction is done to reduce the number of features. Thereafter, the diversity problem between source and destination is solved in order to produce a new feature space and be applicable in the target environment by mapping between features of source and target. Mapped features are adjusted by applying target-environment characteristics to gain a consistent form that represents the target home.

Table 5 shows the differences between two ECAs-IoT that apply machine-learning techniques. Only two architectures were based on machine learning (ML), transfer learning, and distributed learning. Despite a large number of applications of ML algorithms in different domains during the past five years, ML has not been employed heavily to serve ECAs-IoT. The potential of using ML is solving several ECAs-IoT issues is huge, such as in services scheduling, data placement, security, and others.

Table 6 provides a comprehensive comparison among the ECAs-IOT according to different attributes, such as implementation type, focus, and use case. The implementation attribute describes whether the architecture was either implemented in simulation, emulation, testbed, or if it was without an implementation. This helps to investigate further these architectures and setup the best evaluation means when required. The focus attribute describes which IoT issues and tasks addresses by the architecture. The use case attributes provide an insight into the domain the EAC-IoT was used, such as health, smart-city, and others. The year of introducing each architecture is shown in the table as well. The table shows that most ECAs-IoT employ SDN in their architecture, which leads to SDN being a very promising area in IoT networks.

### 5.6. Value of Proposed ECAs-IoT Classifications

On the basis of the previous section, we found that using edge computing alone is not enough, and other techniques or technologies with edge-computing technologies could improve the performance of IoT networks. Next are the most important points learned from the previous section:

- Deploying edge-computing technology to store IoT data in an appropriate node is considered a hot research area to reduce latency, especially for critical IoT applications.
- SDN with edge-computing technology is a hot research area to enhance IoT data placement, because an SDN could act as a centralized controller to the entire network.
- SDNs with edge technology could improve fog computing in the matter of network orchestration.
- SDNs with edge-computing technology can help in making IoT networks more secure.
- Integrating blockchain, SDN, and fog computing is a promising research area.
- Using edge-computing technologies could enhance machine-learning models, such as TTM architectures.

## 6. ECAs-IoT Mapping to IoT 5/3-Layer/ Models

In this section, we map ECAs-IoT to two existing IoT layered models: five- and three-layer models. Mapping includes breaking down each ECA-IoT into its components and matching each component with the corresponding layer in each IoT model whenever applicable. Mapping helps in identifying the capabilities, features, and gaps of every ECA-IoT in terms of its support to IoT layer models.

### 6.1. Existing IoT Layer Models

**Figure 9:**
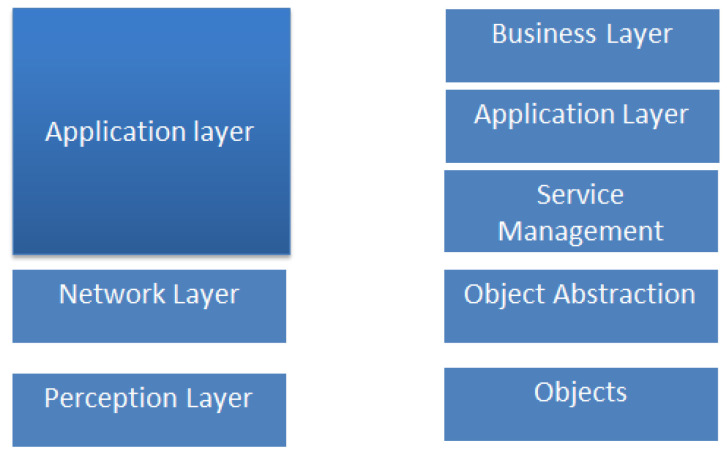
IoT architecture models.

Many IoT reference architectures were proposed, such as IoT-A [148], which is derived from business consideration and the requirement of applications. This survey adopted the reference architecture in [21] which classified IoT architectures into four types of architectures in order to have a reference model for IoT architectures. Figure 9 shows the two popular architectures. The basic model is the three-layer architecture. However, ≈enhancement was done on the three-layer architecture by adding more abstraction layers. This survey adopted these two reference model architectures and analyzed previously mentioned architectures on their basis [21]; the benefit of having a reference model is to define the information type in each layer, which helps researchers to design new protocols. The three-layer architecture consists of the following layers [149]: Perception layer, responsible for gathering information and data from IoT devices and the surrounding environment. Network layer, responsible for transmitting data from the perception layer to the applications layer and data processing before sending them to the application layer. Application layer, which represents the front end of the whole architecture and uses the processed data by the network layer.

The five-layer architecture consists of the following layers:

Object layer, which consists of IoT devices such as sensors and smartphones that are responsible for generating IoT data. Object abstraction layer, responsible for transferring IoT data from the object layer to service-management layer. Service-management layer, which enables programmers to deal with heterogeneous objects by linking services with their requesters. Application layer, which provides high-level services to the customers. Business (management) layer, which manages system activities and services.

### 6.2. Mapping ECAs-IoTs to IoT Layer Models

Table 7 shows a detailed mapping between the surveyed ECAs-IoT and the two common IoT layering models. For each ECA-IoT, we list the components of each architecture, the task of each component, and their corresponding layer(s) in each IoT model if applicable. This mapping helps in multiple ways. For instance, it allows researchers, developers, and designers to quickly grasp the coverage of each ECA-IoT in terms of IoT layers. For instance, the TTM architecture functions at the bottom two layers of the IoT five-layers model, the object abstraction layer, and the objects layer. This immediately implies the lack of support for upper layers such as heterogeneity and management. Additonally, mapping the architecture components to IoT layers helps researchers identify the components to be involved with respect to any improvement or modification they like to make.

Next, the section introduces the two IoT layered models and shows a detailed mapping of the ECAs-IoT to these two models. A more detailed analysis is presented in the next section.

### 6.3. Layer-Mapping Analysis

Mapping allows for quickly identifying the IoT capabilities of each ECA. For instance, some models are only focused on bottom layer functionality, such as TTM and SDNB ECAs. Some ECAs run at all or most of the layers, such as P2A, FSDN, and BSDNV. Some of the ECAs provide services that can only be captured by the five-layer model, such as FSDN, DDA, SDFN, and BSDNV. Most ECAs lack the support of service layers responsible for dealing with heterogeneous objects. Such ECAs rely on the IoT application to handle such issues.

In summary, mapping brings many advantages:

- Allows researchers to identify the capabilities and features of every ECA-IoT in terms of their support to the IoT layered models.
- allows for identifying gaps inside each ECA-IoT in terms of their support of layered IoT models. For instance, when an IoT model layer is not supported by one ECA-IoT, this implies the need to cover that functionality by adding additional components, such as employing an additional protocol inside that ECA-IoT or expecting the IoT application to include that capability.
- Existing IoT layered models do not reference the concept of edge computing. This section connects edge computing with IoT layered models.

## 7. Current ECAs-IoT Limitations

Generally, current ECAs-IoT suffer from technical challenges, such as latency, security challenges, including data privacy and confidentiality, integrity [150], availability, scalability, and network management [107,151,152,153]. This section presents the main challenges that face ECAs-IoT:

### 7.1. Security

Because of the nature of IoT devices and networks [107] IoT security challenges require different mechanisms compared to normal networks. This section discusses the security challenges that face ECAs-IoT that we surveyed in this paper:

#### 7.1.1. Data Confidentiality and Privacy

IoT data may include personal data such as data generated from health sensors. Examples of ECAs-IoT that lack data privacy and confidentiality requirements are discussed here:

- IFogStor [117], iFogStorZ [117], and iFogStorG [118] architectures lack the ability to keep IoT data private because data are stored as plain text in data hosts. A privacy procedure must be performed to protect IoT data from breaches. Some security procedures can be considered, such as adding another layer of security to encrypt confidential data and apply the key management process [154].
- The MFSA [122] architecture focuses on managing task allocation. This architecture has a controller that has the entire knowledge about the whole network. Information that is stored in this controller must be kept secure and not revealed to normal users. Northbound communication should also be encrypted, and applications should be securely coded because any breach in these applications can affect the entire network. Besides, the connection between controller and IoT devices should be encrypted.
- The HDF [133] architecture aims to handle huge amounts of data generated from LSD-IoT, such as smart cities. Data that are generated from smart cities are transmitted to fog nodes to be analyzed. No privacy procedures are taken to preserve user data privacy. To address this limitation, a lightweight encryption algorithm should be applied to encrypt the transmitted data.
- In the HiCH [145] architecture, IoT data are immediately extracted from fog devices without considering IoT data privacy. To handle this issue, the extracted data should be encrypted when transferring data from fog devices.

#### 7.1.2. Data Integrity

Ensuring the accuracy and correctness of IoT data is an important requirement in IoT networks. The following are examples of ECAs-IoT that lack this requirement:

- IFogStor [117], iFogStorZ [117], and iFogStorG [118] do not address the integrity of stored data, and this issue could be handled by adopting the mechanism in [155], a secure way to save private data in the cloud and provide integrity checks to the stored data.
- In the P2A architecture, IoT data privacy is considered; however, the integrity of the data is not. A lightweight constrained application protocol (CoAP) proposed in [156] could be used in communication between IoT devices and fog nodes, handling the integrity of the transmitted message without affecting performance.
- In TTM [136], trained models are transferred without considering the integrity of the trained models. A lightweight integrity protocol should be employed in this architecture, such as the one proposed in [156].

#### 7.1.3. Availability

Keeping resources available is considered a challenge due to the nature of IoT devices and the nature of wireless networks. Next are the examples of ECAs-IoT that lack the availability requirement:

- In IFogStor [117], iFogStorZ [117], and iFogStorG [118] architectures, the data-placement strategy is placed in a preassumed robust node. A procedure must be devised to handle node-failure issues, such as adding a backup controller to avoid the issue of a single point of failure.
- MFSA [76] consists of a controller that has the entire knowledge about the network. A backup procedure must be designed in order to ensure the continuity of allocating tasks, even if the controller is down.

#### 7.1.4. Other Security Challenges

Edge-computing nodes are vulnerable to other security attacks, as follows [157]:

- Sybil attack: this is a type of impersonation attack in which a malicious node pretends to be a legitimate node. This attack could harm IoT networks, because it causes various types of attacks such as the denial of service and breaching personal data [158]. Many solutions were proposed to handle such attacks, such as the algorithm proposed in [159].
- Intrusion detection: a defense mechanism must be insured in ECAs-IoT because any entity can be hacked by external and internal intruders.

### 7.2. Scalability

The capability of a system to increase and cope with the large number and various types of devices should exist in ECAs-IoT. The following are examples of ECAs-IoT that do not scale well:

- IFogStor [117] and iFogstorZ [117] do not scale well in LSD-IoT due to a loss of optimality when the data producer and consumer are not located in the same geographical area. Optimizing resources sharing using clustering resources improves scalability [156].
- The MFSA [76] architecture consists of a controller that knows the entire network. Another layer must be added in order to extend the network orchestration to support LSD-IoT and improve scalability.
- VISAGE [129] is suitable for small- and medium-scale deployment; however, in LSD-IoT, another layer of the controller must be added in order to orchestrate the number of geographical locations.

### 7.3. Management

When managing the huge number of IoT devices, edge-computing nodes, communication among nodes is a burden, unless we employ SDN or network-function-virtualization (NFV) techniques [160]. Numerous challenges come with managing IoT networks:

#### 7.3.1. Data Management

IoT devices produce a tremendous amount of data and managing these data is a challenge [151]. Data-placement-based architectures, such as IFogStor, IFogStorZ, IFogStorM, and IFogStorG, can handle data-management challenges because they place IoT data at appropriate edge nodes, thus reducing data-retrieval latency.

#### 7.3.2. Power Management

IoT devices suffer from limited battery lifetime. Managing when to recharge or replace IoT devices is a challenge.

#### 7.3.3. Device Management

Managing the huge number of IoT devices to ensures that each device is installed and configured properly is a challenge, especially when these devices are installed in unreachable locations. Examples of ESAs-IoT that suffer from this challenge are:

- LSV: this architecture focuses on securing edge devices without re-engineering them, and it does not focus on managing IoT devices.
- TTM: this architecture is an ML-based architecture that focuses on transferring pre-trained models from one location to another. This architecture requires another layer of orchestration to manage the training models in order to avoid outlier model parameters to enhance model accuracy.

#### 7.3.4. Cybersecurity-Management Challenges

This challenge is discussed in the VIA subsection.

### 7.4. Interoperability

Edge-computing devices are owned and controlled by different providers; the common ground is needed to allow for interoperability among all participating entities such as establishing collaborative protocols [66]. An example of ECA-IoT that suffers from this challenge is VISAGE, because any vehicle can act as a fog node, and a collaborative protocol should be established among fog nodes. Many solutions could be adopted to solve these issues, such as using a standard to manufacture product platforms, using the same data format to enhance security, and using this standard in the development of device platforms [161].

### 7.5. Ignorance of Essential Metrics

Some ECAs-IoT focus on certain challenges, but they do not cover important challenges such as the latency of processing IoT data [55], which is a crucial requirement for some critical IoT applications [162]. Next is ECAs-IoT, which suffer from the latency of IoT data processing:

- SIOTOME: although this architecture acts as an IDS for IoT networks, it does not handle data or resource management. Using evolutionary algorithms to optimize IoT service scheduling could enhance latency in IoT networks, such as the solution that was proposed in [44] that employs genetic algorithms to optimize IoT service scheduling.
- P2A: the long process of securing data performed by this architecture introduces extra latency that could impact certain applications.

## 8. IoT Applications in ECAs-IoT

This section provides a taxonomy for IoT applications that require ECAs; also, it covers the main application areas that utilize ECAs. Most of today’s taxonomies consider one or two categories to classify IoT applications. This taxonomy considers five categories in order to classify IoT applications. Current taxonomies did not take into consideration edge-computing devices as the main component in IoT applications; however, this taxonomy takes edge computing as the main part of IoT applications.

### 8.1. IoT Application Taxonomy

**Figure 10:**
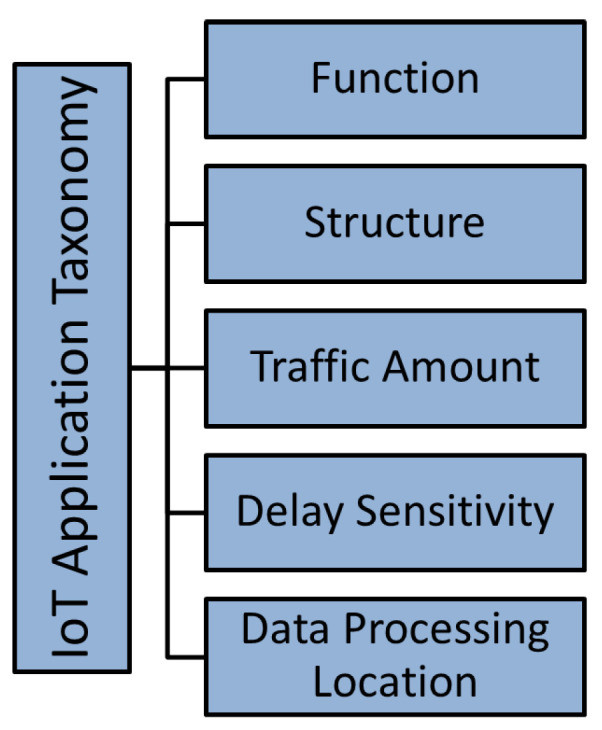
Application category.

This survey presents a taxonomy of IoT applications that is based on surveyed ECAs-IoT applications. The taxonomy (Figure 10) is based on the following categories: IoT application function, the structure of IoT applications, the amount of traffic, sensitivity to delay, sensitivity to different security issues, and whether they require heavy processing on the cloud. The following is the proposed taxonomy.

#### 8.1.1. Function

**Figure 11:**
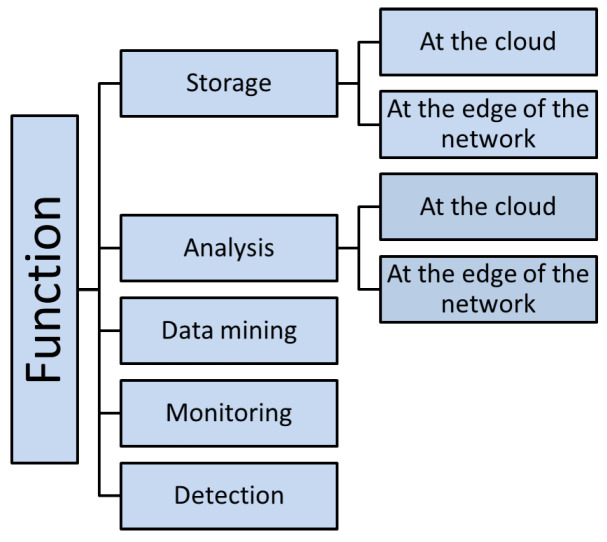
Function category.

This category, as shown in Figure 11, represents the most common functions implemented by the surveyed ECAs-IoT. Categorization is based on how the IoT application deals with IoT data and the functions that it performs such as storing, analyzing, and mining data, and monitoring or applying detection on IoT data. The storage element implies that application functions act as a storage application and whether IoT data are stored at the cloud or the edge. The analysis element represents IoT applications that analyze data while using traditional data-analysis methods, such as statistical algorithms and whether the analysis is done in the cloud or at the edge. The data-mining element covers applications that employ artificial-intelligence techniques such as ML. The next function monitors which IoT applications monitor events, such as heart rates in e-health applications. The last function in this category is the detection function that detects events, such as heart attacks in e-health applications.

Table 8 classifies common IoT applications within this function category, for example, smart industry applications employ more than one function to ensure the quality of manufacturing. However, the green house application acts as a monitor and detector. E-health applications employ all functions in order to provide quality e-health services. Although most applications involve some level of analysis, we also did not list analysis as the main function, unless it was a core function in the application.

Table 9 shows the functions that are employed in each ECA-IoT as in Section 5, whether the ECA-IoT function as storage, data analysis, data mining, event monitoring, or event detection, and shows that most of ECAs-IoT function as data analyzers and employ data-mining techniques in their architectures. This helps IoT application designers to be aware of the functionalities/services available to them by each architecture, so they can select the right architecture for their application from the functionality aspect.

#### 8.1.2. Structure

**Figure 12:**
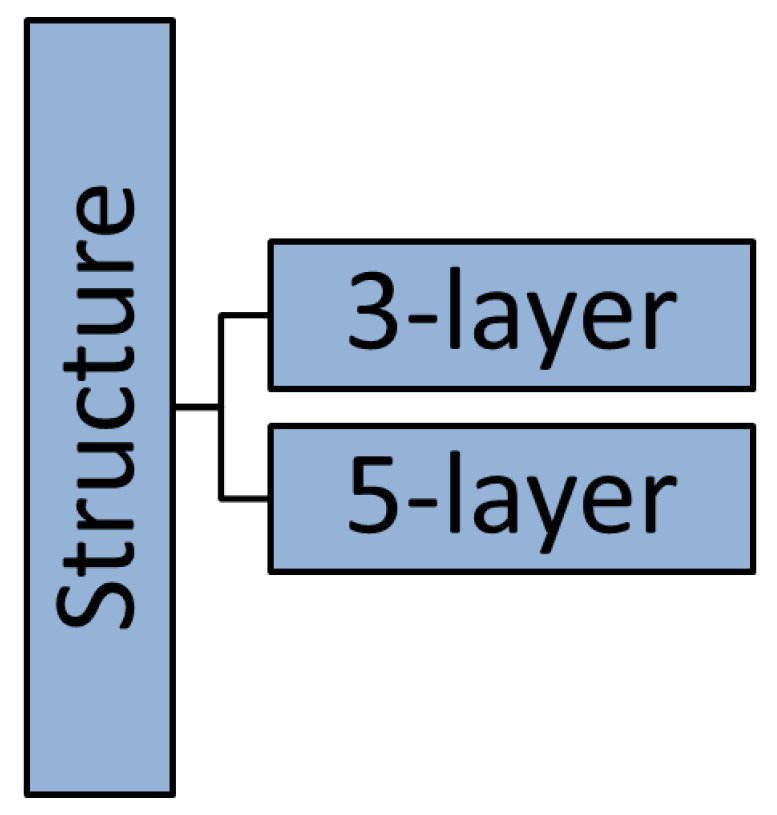
IoT application structure category.

The second category covers the number of layers, and this is discussed thoroughly in Section 6; different IoT applications require different layer functionality. Figure 12 shows the main layered models that we referenced.

#### 8.1.3. Traffic Size

**Figure 13:**
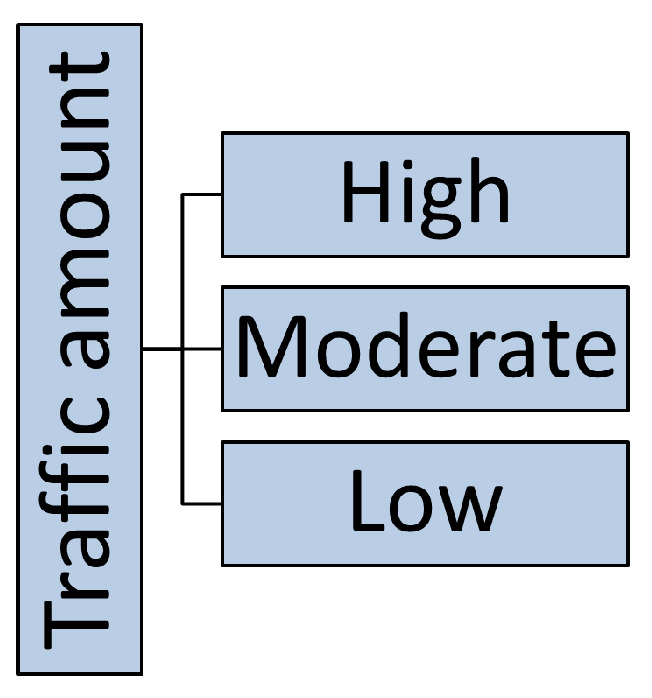
Traffic-amount category.

This category deals with the amount of traffic that is generated from the IoT application as shown in Figure 13. Some applications generate high network traffic, such as smart roads that involve continuous real sensing of data and deliver this to the command center. Other applications perform most of their functions locally and generate minimal outside network traffic, such as smart homes.

Table 10 classifies IoT applications on the basis of generated traffic size by these applications, whether a huge (high), moderate (moderate), or low amount of traffic is generated (low), and it shows that not all of the IoT applications generate the same amount of data.

Table 11 classifies ECAs-IoT on the basis of traffic size generated by each one, whether they are low, moderate, or high, and it shows that most of ECAs-IoT generate high amount of traffic, in which a compression technique could be employed in such architectures. This categorization helps IoT application designers to select the correct ECA-IoT according to network-bandwidth requirements. Hence, according to Table 10 and Table 11, we can suggest MFSA [122] ECA-IoT for, e.g., smart homes.

#### 8.1.4. Delay Sensitivity

**Figure 14:**
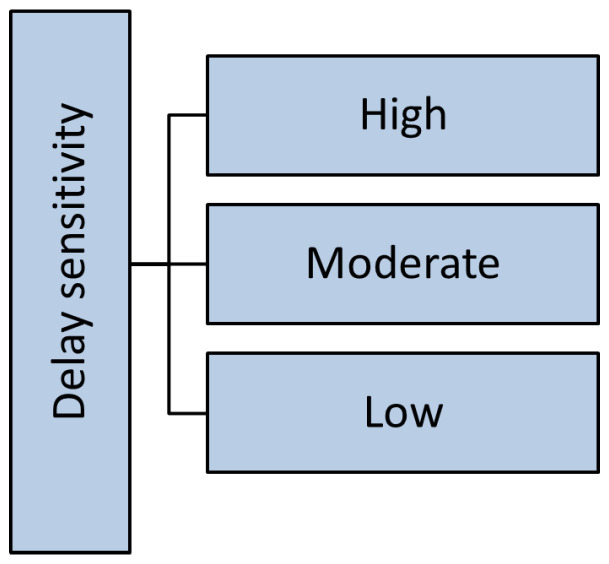
Delay-sensitivity category.

This category classifies IoT applications based on their sensitivity toward delay, as shown in Figure 14. Some applications are of high sensitivity to delay, such as heart-attack-detection applications. Other applications are moderately sensitive to delay, such as smart-home applications, while some applications are less delay-sensitive, such as agricultural applications.

Table 12 classifies IoT applications on the basis of their sensitivity to delay, whether these applications are highly, moderately, or lowly sensitive to delay, and it shows that the same IoT application could be sensitive to delay or not, depending on the case that it handles. However, reducing delay is very important in most IoT applications. Some home applications are not sensitive to delay, such as air conditioning, but home-safety applications are considered to be highly sensitive to delay. E-health applications are sensitive to delay, but greenhouses and smart-lighting applications are not.

Table 13 classifies ECAs-IoT on the basis of the delay of each ECA-IoT, whether it is low, moderate, or high. The table shows that reducing delay is very important in most ECAs-IoT. The ones with high sensitivity toward delay are the ones that generate the least delay. This gives a guideline for IoT application designers and developers to pick the right ECA for their application according to the delay requirements.

#### 8.1.5. Security

**Figure 15:**
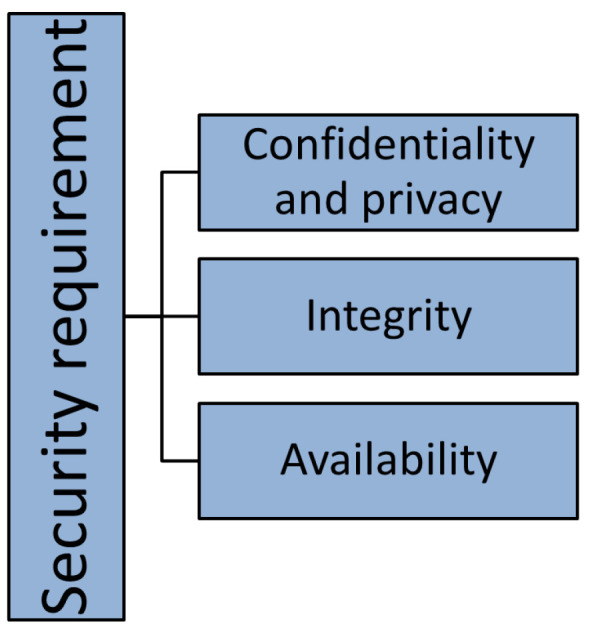
Security-sensitivity category.

This category classifies IoT applications based on their main security requirements when considering the security triad CIA. Confidentiality and privacy are important in some IoT applications, such as those regarding E-health, as shown in Figure 15. Integrity covers IoT applications that are sensitive to data authenticity. Availability covers IoT applications that require the availability of network resources and services.

Table 14 classifies IoT applications on the basis of their security requirements. For example, E-health applications require data confidentiality, integrity, and network availability. However, in greenhouse applications, the minimal security requirement is data integrity. The table shows that integrity and availability requirements are very important.

Table 15 classifies ECAs-IoT on the basis of their security requirements: confidentiality, integrity, and availability. This table shows that all of the security requirements are required in most ECAs-IoT; some ECAs-IoT do not support confidentiality as a built-in feature, and some do not support integrity. Application designers need to consider that and supply the needed requirement within their application if the selected architecture lacks it.

#### 8.1.6. Data Processing

**Figure 16:**
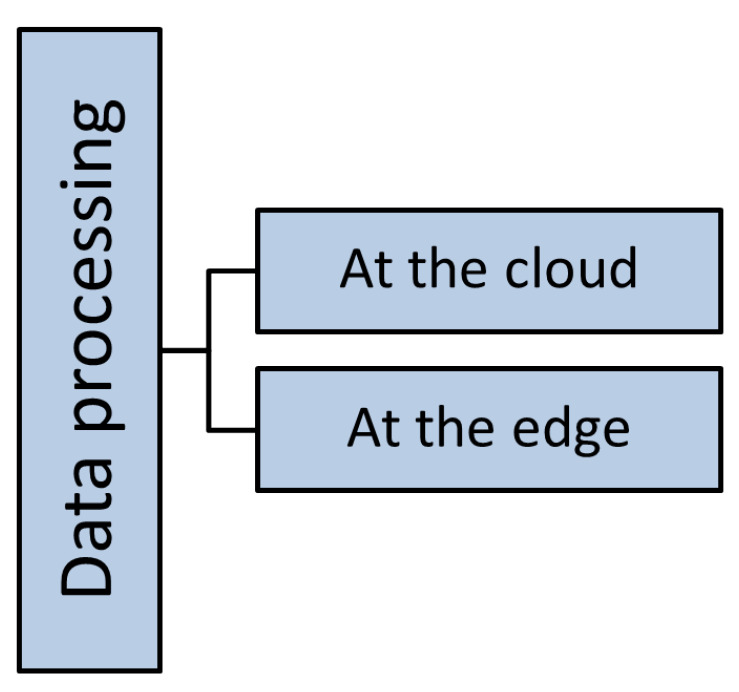
Data-processing category.

This category classifies IoT applications according to whether they require data processing at the cloud or at the edge of the network, as shown in Figure 16. Latency-critical applications require data processing near the end user at the edge of the network, such as e-health and smart-road applications. Edge computing can also reduce bandwidth consumption and enhance latency. Table 16 shows applications that require data processing at the cloud andthe edge of the network, and it shows that the edge plays an important role in processing data near the end user.

Table 17 classifies each ECA-IoT based on the location of data processing, whether it is at the edge or the cloud, and it shows that most of the ECAs-IoT process the data near to the end-user, which reduce bandwidth usage and latency. This helps to match the right architecture with the right application needs in terms of data processing.

### 8.2. ECAs-IoT Applications

This section illustrates some of the IoT applications used by the surveyed architectures and the suitability of the ECAs-IoT to certain IoT applications.

#### 8.2.1. Smart City

This subsection lists ECAs-IoT that serve or simulate smart-city applications:

- IFogStor [117], IFogStorZ [117], and IFogStorM [119] were simulated using smart-city use cases. A generic smart-city use case was considered in which different types of sensors generated and sent data to IoT applications installed over fog nodes and data centers. The infrastructure consisted of sensors, fog nodes, and data centers. GW, LPoP, and RPoP were considered to be fog nodes organized hierarchically. Sensors collected and generated data from a real-world environment, and then sent it to an application instance installed in the fog node located in GW. Subsequently, each application instance sent the result of processing data to one or more application instance(s) located in LPoP based on the number of data consumers and producers. Thereafter, LPoP sent the result of processing data to application instances installed in RPoP and data centers. Lastly, data centers stored processed data for archiving IoT data. The Ifogsim tool was used for simulation. The dataset used for simulation was generated from sensors.
- IFogStorG [118]: this architecture is an enhancement of IFogStorZ [117]. The difference here is the used graph-partitioning technique. Ifogsim was used to generate the infrastructure.
- HDF [133] focuses on pipeline systems in smart cities; the used dataset was real-time by building a real prototype of the pipeline system.
- LSV [137] was applied to several IoT applications such as smart cities, e-health, and smart-home applications to prove that the architecture works on all types. The experimental results showed that the LSV architecture improved system service efficiency and ensured data integrity.

#### 8.2.2. Smart Home

This subsection lists ECAs-IoT that were designed to serve smart-home applications:

- SIOTOME [139], which aims to enhance threat and vulnerability detection in smart-home applications.
- TTM [147] focuses on transferring pretrained smart-home applications to other smart-home applications to benefit from pretrained model knowledge.

#### 8.2.3. E-Health

This subsection lists ECAs-IoT that were designed to serve e-health applications:

- P2A [136]: focuses on preserving e-health data while aggregating them. The data used to evaluate the system architecture are the MHEALTH dataset [136], which consists of one million records that were generated from 24 sensors with 24 signals. In evaluation, only one signal was used to evaluate the architecture.
- HiCH [145]: aims to perform continuous health monitoring by applying machine-learning techniques. The case study focused on arrhythmia detection for patients suffering from cardiovascular diseases (CVDs). The used dataset was the “long-term ST dataset“ available on [163,164].

#### 8.2.4. Intertransportation System

This subsection lists the ECAs-IoT that were designed to serve inter-transportation systems:

- VISAGE [129] focuses on orchestrating inter-transportation systems to benefit from vehicles as fog nodes and employ cloud centers as SDN controllers to control the entire system. No simulation was performed in order to test this architecture.

## 9. Recommendations and Future Work

Based on the study of ECAs-IoT, we recommend four future directions for researchers and IoT application designers in selecting an ECA for their IoT application.

### 9.1. Use of Existing ECAs-IoT for New Scenarios

Researchers and application designers can use an ECA-IoT without modification for an IoT application that is different from the one for which the ECA-IoT was designed. Below are examples of using existing ECAs-IoT for different IoT applications.

- SIOTOME [139] is suitable for smart cities because it helps in detecting vulnerabilities in LSD-IoT because this architecture acts like an IDS system. The IDS is also up-to-date, because this architecture employs ML techniques to update it. Besides, this architecture is based on SDN technology that can orchestrate the heterogeneous nature of the network.
- IFogStorZ [117] and IFogStorG [118] enhance intertransportation systems because they enhance the latency requirement, which is a major requirement in intertransportation systems.
- MFSA [122] reduces the required cost to allocate tasks to appropriate nodes, and this enhances inter-transportation systems.
- MAFECA [123] enhances task assignment, which is mandatory in inter-transportation systems.
- SBDC [138] acts as an IDS system in an IoT network. This architecture could enhance inter-transportation systems by handling services while resisting IoT attacks.
- TTM [147] is recommended for inter-transportation systems, because the nature of transportation systems requires more knowledge than what could be attained from pre-trained models.
- SDNDB [142] is a security SDN and blockchain-based architecture that securely enhances latency. Latency and security are important requirements in inter-transportation systems.
- The P2A [136] architecture could preserve privacy for home applications that usually deal with confidential data.

Table 18 summarizes this section by illustrating existing ECAs-IoT and potential IoT applications that they can serve.

#### 9.1.1. Revised ECA-IoT

A second option for researchers and application designers is to modify an existing ECA-IoT to make it suitable for IoT applications different from the ones for which it was originally designed. Below is an example of this option:

- TTM [147] uses a transfer-learning aspect to transfer intelligence from one system to another. Therefore, using this architecture is very useful in smart cities to make IoT applications more aware of new and rare incidents by transferring intelligence and trained models from one city to another. However, another layer of security should be added in order to ensure the authenticity of transferred models and to prevent intruders from modifying the transferred models.

#### 9.1.2. Hybrid ECA-IoT

The third option is merging two or more ECAs-IoT to provide new capabilities for IoT applications:

- IFogStorG [118] and SIOTOME [139] give us a secure architecture that handles data management. The SIOTOME part detects early threats from IoT devices, and IFogStorG is responsible for managing IoT data.
- E-health applications require an architecture that provides the following functions: data analysis, monitoring, detection, latency, data privacy, integrity, and network availability. In order to improve e-health applications, using a hybrid architecture can enhance the quality of service of e-health applications. This hybrid architecture includes SDNDB, because it handles the required functionalities of the e-health application, IFogStorM handles the latency requirement, and the P2A application ensures E-health data privacy.

#### 9.1.3. New ECA-IoTs

The last option is to build a new ECA-IoT. In the case of an IoT application that has complex requirements that cannot be met by any of the previous three options—existing, modified, and merged—a new ECA-IoT is required. The new proposed architecture can still benefit from the design aspect of existing ones and offer similar components, layers, and services.

## 10. Conclusions

With its ability of processing data near end-users, which is a major demand for IoT applications, and especially time-critical ones, edge-computing technology is becoming an attractive option.

This survey classified ECAs-IoT according to IoT challenges that they aim to handle. This includes data-placement-based architectures that aim to handle IoT data management, big-data-analysis-based architectures that aim to handle the analysis of IoT big data, security-based architectures that focus on securing IoT networks, machine-learning-based architectures that provide ML services in IoT networks, and orchestration-based architectures that employ several techniques, such as SDN, to handle management issues in IoT networks. Besides, this survey classified ECAs-IoT based on two reference models, three- and five-layer architectures. Additionally, this survey mapped ECAs-IOT into two existing IoT layer models, which help identify the capabilities, features, and gaps of every architecture.

This paper also classified IoT applications that are based on the application that the architecture serves. Another contribution is recommending one or more ECAs-IoT for IoT applications based on the application requirements. This is manifested in mapping between ECAs-IoT and common IoT applications in general. This paper included the main challenges that ECAs-IoT faces, such as security, scalability, and management. This survey concluded with the ability to use ECAs-IoTs for IoT applications in four different scenarios, the use of existing ECAs, or modifying one to better fit the requirements of a certain application, merging two or more ECAs, and developing an entirely new ECA as a last resort.

## Figures and Tables

**Table 1:** Survey-comparison table.

Survey Paper	IoT Arch	IoT Apps	IoT Chall	IoT Tech	FC Chall	FC-IoT Apps	FCA-IoT	FC-IoT Challenges	FC-Algo	FC-IoT Platforms
Maier in [95]		✔								
Al-Fuqaha et al. in [21]		✔								
Pflanzner et al. [96]		✔								
Asghari et al. [98]		✔								
Ray [99]	✔									
Razzarue et al. [100]				✔						
Lao et al. in [101]		✔								
Lin et al. [94]	✔		✔	✔		✔				
Farahzadi et al. [102]	✔			✔						
Sethi et al. [103]	✔			✔						
Atzori et al. [97]		✔		✔						
Abbas et al. [104]		✔	✔							
Miorandi et al. [105]		✔	✔	✔						
Dastjerdi et al. [106]						✔				
Mahmud et al. [107]					✔					
Mouradian et al. [109]					✔	✔	✔		✔	
Atlam et al. [110]						✔		✔		
Bellavista et al. [111]			✔			✔				
Puliafito et al. [112]					✔	✔	✔			✔

**Table 2:** Comparison of data-placement architectures.

Architecture	Deployed Techniques	Improvement	Weakness
IFogStor [117]	Exact solution	Latency	Not suitable for LSD-IoT
IFogStorZ [117]	Divide and concur	Latency	Loss of optimally occurs
IFogStorG [118]	Graph partitioning and Floyd’s algorithm strategy	Latency	Not simple to implement
IFogStorM [119]	Greedy algorithms	Latency	Network overhead in LSD-IoT

**Table 3:** Comparison between management-based architectures to manage an IoT network.

Architecture	Technique	Enhancement	Weakness
VISAGE [129]	Clustering, multilevel SDN and 5G	Network orchestration	Does not fit LSD-IoT
DDA [132]	SDN and cloudlet	Reducing latency and big data analysis	-
FSDN [130]	SDN	Network orchestration	No resource management
SDFN [131]	SDN and fog computing	Network orchestration	No centralized control of the entire network
MFSA [122]	Integer-programming formulation	Minimize cost of service allocation	Not suitable for large-scale deployment
MAFECA [123]	Multiagent framework	Task assignment between cloud and edge devices	Affected by environment-adaptation ability, not suitable for large-scale deployment, and difficult to dynamically assign tasks
HAM [124]	Workload-placement algorithm	Enhances service allocation for small- and large-scale employment	-
SAT [125]	Transparent computing	Enhances scalability and reduce response tome	Heterogeneity
E-ALPHA [126]	-	Enhances scalability and interoperability	Heterogeneity

**Table 4:** Comparison of ECAs-IoT security

Architecture	Technique	Enhancement	Weakness
P2A [136]	Machine-learning techniques	Sensory-data privacy	Does not consider data integrity
LSV [137]	Embedded virtualization and trust mechanisms	Secure edge devices without re-engineering IoT applications	Vulnerable to run-time attacks
SBDC [138]	Trust mechanisms and service templates	Data integrity and service efficiency	Not suitable for LSD-IoT
SIOTOME [139]	IDS and machine-learning techniques	Threat and vulnerability detection	-
SDNDB [142]	SDN and blockchain	Reducing latency in a secure manner and enhancing security	-
MCS [140]	-	Reducing privacy threats and enhancing latency	Interoperability
ECV [141]	VID	Latency, privacy, and integrity	Does not fit LSD-IoT
BSDNV	Blockchain and SDN	Trust within system components	Not supporting big-data analysis

**Table 5:** Comparison of ECAs-IoT based on machine learning (ML).

Architecture	Technique	Enhancement	Weakness
HiCH [145]	MAPE-K models	Latency and response time	-
TTM [147]	Embedded virtualization and trust mechanisms	Secure edge devices	Vulnerable to runtime attacks

**Table 6:** A comprehensive comparison among ECAs-IoT.

Architecture	Techniques	Implementation	Focus	Use case	Year
IFogStor [117]	Exact solution	Simulation	Data placement	Smart city	2017
IFogStorZ [117]	Divide and conquer, heuristic approach	Simulation	Data placement	Smart city	2017
IFogStorG [118]	Divide and conquer, graph theory	Simulation	Data placement	Smart city	2018
IFogstorM [119]	greedy algorithm	Simulation	Data placement	Smart city	2019
MFSA [122]	Integer program	Simulation	Service allocation	Real-world scenarios	2017
MAFECA [123]	Multiagent framework	Simulation	Task assignment between cloud and edge devices	e-health	2018
HAM [124]	Workload placement algorithm	simulation	network management	smart city	2016
SAT [125]	Transparent computing	emulation	Network orchestration	E-health	2017
E-ALPHA [126]	-	Simulation	Network management	E-health	2020
VISAGE [129]	Clustering, multilevel SDN, and 5G	No implementation	Network orchestration	VANET	2018
FSDN [130]	SDN	No implementation	Resource management	VANET	2015
SDFN [131]	SDN and fog computing	-	Orchestrate the network	Intertransportation system, video surveillance, and precision agriculture	2017
DDA [132]	SDN	Testbed	Latency, big data analysis	Big-data analysis (video analytics)	2018
HDF [133]	Hidden Markov model	Simulation	Big-data analysis	Pipeline system	2015
P2A [133]	Machine learning	Testbed and Simulation	Privacy preserving	E-health	2018
LSV [137]	Embedded virtualization and trust mechanisms	Simulation	Secure edge devices without re-engineering IoT application	Smart city	2018
SBDC [138]	Trust mechanisms and service templates	Simulation	Data integrity and services efficiency	Smart transportation system	2018
SIOTOME [139]	IDS and machine-learning techniques	-	Threat and vulnerability detection	Smart home	2018
MCS [140]	-	Simulation	Privacy and latency	Mobile crowd sensing	2017
ECV [141]	VID	Simulation	latency, privacy, and integrity	Smart city	2017
SDNDB [142]	SDN and blockchain	Testbed	Network orchestration and security	No application	2017
BSDNV [144]	SDN and blockchain	Simulation	Enhances trust in networking platforms	Intertransportation system	2019
HiCH [145]	MAPE-K model	Simulation	Latency and response time	e-health	2017
TTM [147]	Feature analysis, hidden Markov model and machine learning	Testbed	Simulation	Smart home	2018

**Table 7:** ECAs-IoT mapping to IoT layered models.

ECA Name	Architecture Component	Task Done by Component	Corresponding 3-Layer (Model Layer)	Corresponding 5-Layer (Model Layer)
IFogStorZ	Sensors	Sensing the environment	Layer 1	Layer 1
	Higher-level application instances	Offer a higher level of services	Layer 3	Layer 4
IFogStorG	Sensors	Collect data from the environment	Layer 1	Layer 1
	GW	Responsible for transferring data	Layer 2	Layer 2
	Application instance	Processes the incoming requests	Layer 3	Layer 4
IFogStorM	Sensors	Collect data from the environment	Layer 1	Layer 1
	GW	Responsible for transferring data	Layer 2	Layer 2
	Fog nodes	Provide services to local geographical area	Layer 3	Layer 4
MFSA	IoT devices	Collect data from the environment	Layer 1	Layer 1
	GW	Responsible for transmission	Layer 2	Layer 2
	controller	Controls the entire network	Layer 2	Layer 5
MAFECA	IoT devices and sensors	Sensing the environment	Layer 1	Layer 1
	fog nodes	Provide application services	Layer 3	Layer 4
VISAGE	Mobile devices and sensors	Sensing the environment	Layer 1	Layer 1
	Base stations	Responsible for connectivity	Layer 2	Layer 2
	LSDNC and CSDNC	Controlling the network	Layer 2	Layer 5
	Vehicles	Act as fog nodes that provide services to end users	Layer 3	Layer 4
FSDN	Vehicles	Acting as sensors to sense the environment	Layer 1	Layer 1
	Base stations	Responsible for connectivity	Layer 2	Layer 2
	RSUC	Responsible for controlling on a group of RSUs	Layer 2	Layer 5
	RSUs	Act as fog nodes that provide services to end-users	Layer 3	Layer 4
	SDN controller	Responsible for managing the entire network	NA	Layer 5
SDFN	IoT devices	Responsible for collecting data from the environment	Layer 1	Layer 1
	SDN controller	Responsible for managing the entire network	NA	Layer 5
DDA	IoT devices and sensors	Sensing the environment	Layer 1	Layer 1
	DCs	Connectivity and monitoring bandwidth flow	Layer 2	Layer 2
	DC controller, TSDNO, and GSO	Orchestrating the network	NA	Layer 5
SDNB	Mobile devices and sensors	Sensing the environment	Layer 1	Layer 1
	Base stations	Responsible for wireless communication and acting as a forwarding plan for the SDN controller	Layer 2	Layer 2
	SDN controller	Responsible for providing programming interfaces to network management operators	NA	Layer 2
HDF	Sensors	Sensing the environment and provide timely analysis for IoT data	Layer 1 and Layer 3	Layer 1 and Layer 4
	Group of edge devices	Responsible for covering a small group of sensors	Layer 3	Layer 3
P2A	Sensors	Sensing the environment	Layer 1	Layer 1
	GW	Transmitting media	Layer 2	Layer 2
	Fog nodes	Answering queries	Layer 3	Layer 4
	Fog centers	Processing queries	Layer 3	Layer 4
	Cloud servers	Responsible for the aggregation process	Layer 3	Layer 4
HiCH	Sensors	Collect data from the environment	Layer 1	Layer 1
	System management component	Transmit data	Layer 2	Layer 2
	Execute part	Sending updates to parts	Layer 3	Layer 4
LSV	IoT devices	Collect data from the environment	Layer 1	Layer 1
	Secured edge devices	Provide secured edge applications without reengineering them	Layer 3	Layer 4
SBDC	IoT devices	These devices are vulnerable to attacks	Layer 1	Layer 1
	Edge platform	Establish services templates	Layer 2	Layer 3
SIOTOME	Smart Home sensors	Collect data from the environment	Layer 1	Layer 1
	GWs	Provides connectivity between smart home sensors with ISP	Layer 2	Layer 2
	Edge analyzer	Analyse data for further analysis	Layer 2	Layer 3
	Cloud controller	Collecting reports and control the communication	Layer 2	Layer 5
ECV	IoT devices	Generate IoT data	Layer 1	Layer 1
	Proxy servers	Responsible for connectivity	Layer 2	Layer 2
	Data validation item	Responsible for security	Layer 2	Layer 3
	Virtual IoT devices	Process, validate, and annotate IoT data	Layer 2	Layer 3
BSDNV	Smart Vehicles	Collect data from the environment	Layer 1	Layer 1
	RSUs and base stations	Responsible for connectivity	Layer 2	Layer 2
	Fog nodes	Provide services to vehicles	Layer 3	Layer 4
	RSUH	Controls the overhead between RSUs and vehicles	NA	Layer 5
	SDN controller	Controls the entire network	NA	Layer 5
TTM	Sensors	Collect data from the environment	Layer 1	Layer 1
	Edge nodes	Transfer trained to other edge nodes	Layer 2	Layer 2

**Table 8:** Classification of IoT applications within application function category.

App	Storage	Analysis	Data Mining	Monitoring	Detection
Smart home		✔		✔	✔
Smart lighting				✔	
Smart road				✔	✔
Smart industry	✔	✔	✔	✔	✔
Green house				✔	✔
E-health	✔	✔	✔	✔	✔

**Table 9:** Classification of ECAs-IoT within application function category.

App	Storage	Analysis	Data mining	Monitoring	Detection
IFogStor [117]	✔				
IFogStorZ [117]	✔				
IFogStorG [118]	✔				
IFogstorM	✔				
MFSA [122]				✔	
MAFECA [123]			✔		
VISAGE [129]		✔		✔	✔
FSDN [130]		✔		✔	✔
SDFN [131]	✔	✔	✔	✔	✔
DDA [132]		✔			
HDF [133]		✔			
P2A [133]	✔	✔			
LSV [137]		✔			
SBDC [138]			✔		
SIOTOME [139]		✔			
ECV [141]		✔	✔	✔	
SDNDB [142]		✔	✔		✔
BSDNV [144]		✔	✔	✔	✔
HiCH [145]		✔	✔		
TTM [147]			✔		

**Table 10:** Classification of IoT applications within traffic-amount category.

App	Low	Moderate	High
Smart home	✔		
Smart lighting	✔		
Smart road			✔
Smart industry			✔
Green house	✔		
E-health		✔	

**Table 11:** Classification of ECAs-IoT within traffic-size category.

App	Low	Moderate	High
IFogStor [117]			✔
IFogStorZ [117]			✔
IFogStorG [118]			✔
IFogstorM			✔
MFSA [122]	✔		
MAFECA [123]	✔		
VISAGE [129]			✔
FSDN [130]			✔
SDFN [131]		✔	
DDA [132]			✔
HDF [133]			✔
P2A [133]	✔		
LSV [137]		✔	
SBDC [138]			✔
SIOTOME [139]			✔
ECV [141]			✔
SDNDB [142]		✔	
BSDNV [144]			✔
HiCH [145]		✔	
TTM [147]			✔

**Table 12:** Classification of IoT applications within delay-sensitivity category.

App	Low	Moderate	High
Smart home		✔	✔
Smart lighting	✔		
Smart road			✔
Smart industry			✔
Green house	✔		
E-health			✔

**Table 13:** Classification of ECAs-IoT and sensitivity-to-delay category.

App	Low	Moderate	High
IFogStor [117]			✔
IFogStorZ [117]			✔
FogStorG [118]			✔
IFogstorM			✔
MFSA [122]	✔		
MAFECA [123]			✔
VISAGE [129]			✔
FSDN [130]			✔
SDFN [131]			✔
DDA [132]		✔	
HDF [133]		✔	
P2A [133]		✔	
LSV [137]	✔		
SBDC [138]			✔
SIOTOME [139]		✔	
ECV [141]		✔	
SDNDB [142]	✔		
BSDNV [144]			✔
HiCH [145]			✔
TTM [147]	✔		

**Table 14:** The classification of IoT applications and security requirement.

ECA-IoT	Confidentiality and Privacy	Integrity	Availability
Smart home		✔	✔
Smart lighting			
Smart road	✔	✔	✔
Smart industry		✔	✔
Green house		✔	
E-health	✔	✔	✔

**Table 15:** Classification of ECAs-IoT within security-requirement category.

App	Confidentiality	Integrity	Availability
IFogStor [117]	✔	✔	✔
IFogStorZ [117]	✔	✔	✔
IFogStorG [118]	✔	✔	✔
IFogstorM	✔	✔	✔
MFSA [122]	✔		
MAFECA [123]			✔
VISAGE [129]	✔	✔	✔
FSDN [130]	✔	✔	✔
SDFN [131]	✔	✔	✔
DDA [132]		✔	
HDF [133]		✔	✔
P2A [133]	✔	✔	✔
LSV [137]		✔	
SBDC [138]	✔	✔	✔
SIOTOME [139]			✔
ECV [141]		✔	
SDNDB [142]	✔		
BSDNV [144]	✔	✔	✔
HiCH [145]	✔	✔	✔
TTM [147]	✔		

**Table 16:** Classification of IoT applications within data-processing-location category.

App	At the Edge	At the Cloud
Smart home	✔	✔
Smart lighting	✔	
Smart road	✔	✔
Smart industry	✔	✔
Green house	✔	
E-health	✔	✔

**Table 17:** Classification of ECAs-IoT within data-processing location category.

ECAs-IoT	At the Edge	At Cloud
IFogStor [117]	✔	
IFogStorZ [117]	✔	
IFogStorG [118]	✔	
IFogstorM	✔	
MFSA [122]	✔	
MAFECA [123]	✔	
VISAGE [129]	✔	✔
FSDN [130]	✔	✔
SDFN [131]	✔	✔
DDA [132]	✔	✔
HDF [133]	✔	✔
P2A [133]	✔	✔
LSV [137]	✔	✔
SBDC [138]	✔	✔
SIOTOME [139]	✔	✔
ECV [141]	✔	
SDNDB [142]	✔	✔
BSDNV [144]	✔	
HiCH [145]	✔	
TTM [147]	✔	

**Table 18:** Use of existing ECAs-IoT for other IoT applications.

Architecture	Smart Cities	Intertransportation Systems	Smart Home	E-Health
MFSA [122]	✔	✔		
MAFECA [123]	✔			
SIOTOME [139]	✔			
TTM [147]	✔	✔		
IfogstorG [118]		✔		
IfogstorZ [117]		✔		
SBDC [138]		✔		
P2A [136]			✔	
SDNB [142]				✔
IFogStorM [119]				✔

## Abbreviations

The following abbreviations are used in this manuscript:

IoT	Internet of Things
SDN	Software-defined network
ML	Machine learning
RFID	Radio-frequency identification
WSN	Wireless-sensor network
LSD-IoT	Large-scale IoT deployments
MEC	Mobile edge computing
Apps	Applications
GW	Gateway
LSDNC	Local SDN controller
CSDNC	Central SDN controller
DC	Data center
RSU	Road-side units
TSDNO	Transport SDN controller
GSO	Global service orchestrator
NA	Not applicable
